# Blockade of αvβ6 and αvβ8 integrins with a chromogranin A-derived peptide inhibits TGFβ activation in tumors and suppresses tumor growth

**DOI:** 10.1186/s13046-025-03352-4

**Published:** 2025-03-08

**Authors:** Anna Maria Gasparri, Arianna Pocaterra, Barbara Colombo, Giulia Taiè, Chiara Gnasso, Alessandro Gori, Federica Pozzi, Andrew Smith, Fulvio Magni, Alessia Ugolini, Matteo Doglio, Maria Chiara Bonini, Anna Mondino, Angelo Corti, Flavio Curnis

**Affiliations:** 1 Tumor Biology and Vascular Targeting Unit, Division of Experimental Oncology, IRCCS San Raffaele Scientific Institute, Milan, Italy; 2 Lymphocyte Activation Unit, Division of Immunology, IRCCS San Raffaele Scientific Institute, Milan, Italy; 3 Experimental Imaging Center, IRCCS San Raffaele Scientific Institute, Milan, Italy; 4 Istituto di Scienze e Tecnologie Chimiche (SCITEC-CNR), National Research Council of Italy, Milan, Italy; 5 Department of Medicine and Surgery, Clinical Proteomics and Metabolomics Unit, University of Milano-Bicocca, Milan, Italy; 6 Experimental Hematology Unit, Division of Immunology, IRCCS San Raffaele Scientific Institute, Milan, Italy; 7 Vita-Salute San Raffaele University, Milan, Italy

**Keywords:** αvβ6- and αvβ8-integrins, Transforming growth factor-β (TGFβ), Albumin, Chromogranin A, Cancer, S-NGR-TNF

## Abstract

**Background:**

The αvβ6- and αvβ8-integrins, two cell-adhesion receptors upregulated in many solid tumors, can promote the activation of transforming growth factor-β (TGFβ), a potent immunosuppressive cytokine, by interacting with the RGD sequence of the latency-associated peptide (LAP)/TGFβ complex. We have previously described a chromogranin A-derived peptide, called “peptide **5a**”, which recognizes the RGD-binding site of both αvβ6 and αvβ8 with high affinity and selectivity, and efficiently accumulates in αvβ6- or αvβ8-positive tumors. This study aims to demonstrate that peptide **5a** can inhibit TGFβ activation in tumors and suppress tumor growth.

**Methods:**

Peptide **5a** was chemically coupled to human serum albumin (HSA) to prolong its plasma half-life. The integrin recognition properties of this conjugate (called **5a**-HSA) and its capability to block TGFβ activation by αvβ6^+^ and/or αvβ8^+^ cancer cells or by regulatory T cells (Tregs) were tested in vitro. The in vivo anti-tumor effects of **5a**-HSA, alone and in combination with S-NGR-TNF (a vessel-targeted derivative of tumor necrosis factor-a), were investigated in various murine tumor models, including pancreatic ductal adenocarcinoma, fibrosarcoma, prostate cancer, and mammary adenocarcinoma.

**Results:**

In vitro assays showed that peptide **5a** coupled to HSA maintains its capability of recognizing αvβ6 and αvβ8 with high affinity and selectivity and inhibits TGFβ activation mediated by αvβ6^+^ and/or αvβ8^+^ cancer cells, as well as by αvβ8^+^ Tregs. In vivo studies showed that systemic administration of **5a**-HSA to tumor-bearing mice can reduce TGFβ signaling in neoplastic tissues and promote CD8-dependent anti-tumor responses. Combination therapy studies showed that **5a**-HSA can enhance the anti-tumor activity of S-NGR-TNF, leading to tumor eradication.

**Conclusion:**

Peptide **5a** is an efficient tumor-homing inhibitor of αvβ6- and αvβ8-integrin that after coupling to HSA, can be used as a drug to block integrin-dependent TGFβ activation in tumors and promote immunotherapeutic responses.

**Graphical Abstract:**

**Figure:**
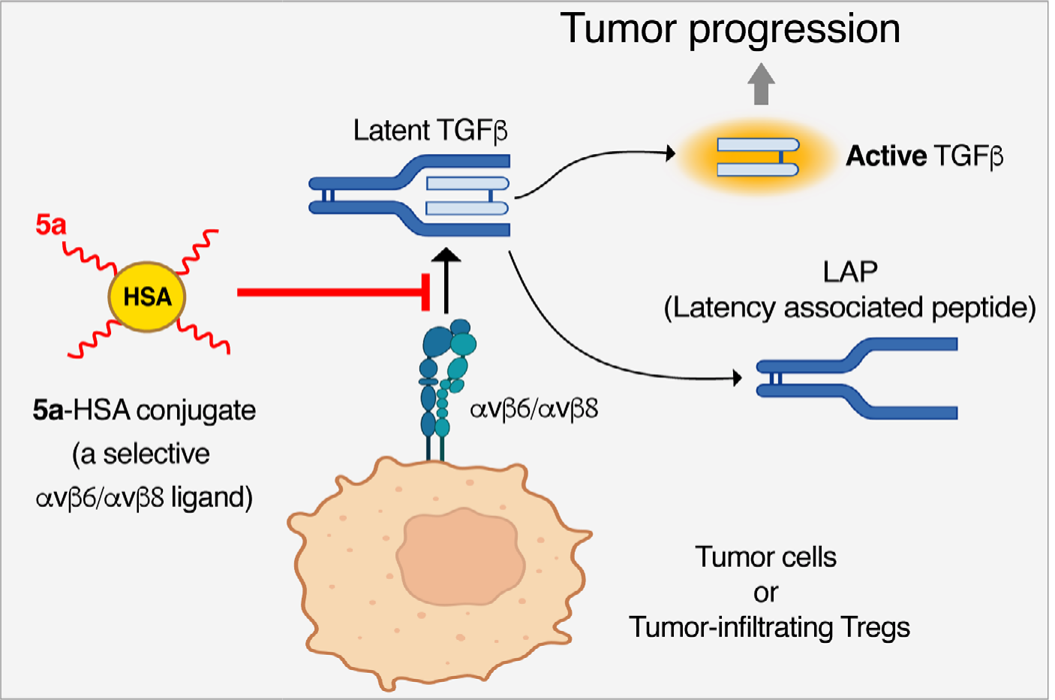
The **5a**-HSA conjugate, a compound consisting of the chromogranin A-derived peptide **5a** coupled to human serum albumin (HSA), can bind the RGD binding site of αvβ6 and αvβ8 integrins expressed by tumor cells and tumor-infiltrating regulatory T cells (Tregs) and inhibits αvβ6- and/or αvβ8-mediated activation of TGFβ, thereby reducing its immunosuppressive effects and promoting anti-tumor immune responses

**Supplementary Information:**

The online version contains supplementary material available at 10.1186/s13046-025-03352-4.

## Background

A growing body of evidence suggests that αvβ6- and αvβ8-integrins, two cell-adhesion receptors upregulated in many tumors, represent potential targets for tumor imaging and therapy. For example, integrin αvβ6 is overexpressed in pancreatic ductal adenocarcinoma (PDAC), head and neck squamous cell carcinoma, colon, liver, breast, and ovarian cancers, among others [1–7], with important prognostic implications [1, 4, 8–10], whereas αvβ8 is overexpressed in various carcinomas (such as ovarian, skin, uterine endometrioid, breast ductal, gastric adenocarcinoma, and head-and-neck squamous cell carcinomas) and other cancer types (including melanomas and glioblastomas) [11–14] and by tumor-infiltrating regulatory T cells (T_regs_) [11, 15, 16]. Thus, compounds capable of targeting these integrins could be used, in principle, for delivering imaging and therapeutic agents to tumors. Furthermore, considering that both αvβ6 and αvβ8 have high affinity for the RGD sequence of the TGFβ1 latency-associated peptide (LAP) and participate in the activation of TGFβ (a potent immunosuppressive cytokine) [5, 7, 15] compounds capable of interfering with such interactions may reshape the immunosuppressive microenvironment in tumors and unleash anti-tumor activities. According to this view, it has been reported that anti-αvβ6 antibodies can inhibit the growth of αvβ6-positive tumors through a TGFβ-regulated mechanism [17], whereas other studies have shown that antibodies against αvβ8 can inhibit the activation of TGFβ in murine tumors and induce durable anti-tumor immunity [11, 18]. Bi-specific compounds that target the LAP/TGFβ complex-binding site of both αvβ6 and αvβ8 integrins may represent, therefore, not only useful ligands for drug delivery to tumors but also important tumor-homing inhibitors of TGFβ-mediated immunosuppressive mechanisms.

We have previously described a chromogranin A-derived peptide, called “peptide **5a”**, which selectively recognizes the RGD-binding site of both αvβ6 and αvβ8 with high affinity and selectivity [19, 20]. This peptide (CFETLRGDLRILSILR**X**_**1**_QNL**X**_**2**_KELQD) contains the canonical RGDLXXL αvβ6-integrin recognition motif, followed by an amphipathic α-helix chemically stabilized by a triazole bridge between the propargylglycine (**X**_**1**_) and azidolysine (**X**_**2**_) residues. This peptide efficiently accumulates in αvβ6- or αvβ8-positive tumors, including pancreatic and prostate cancer models [19, 20].

This study intends to evaluate the potential of this peptide as a drug to inhibit TGFβ activation in tumors. To this end, we coupled peptide **5a** to human serum albumin (HSA), to increase the peptide plasma half-life and tested its ability to block TGFβ activation in various in vitro and in vivo tumor models and induce anti-tumor effects. We show that this conjugate (called **5a**-HSA) can efficiently bind αvβ6^+^ and/or αvβ8^+^ cancer cells as well as αvβ8^+^ immunosuppressive Tregs, and, consequently, inhibits TGFβ activation by these cells *in vitro.* Furthermore, we demonstrate that **5a**-HSA can reduce TGFβ activation in tumors and induce strong anti-tumor effects in various in vivo murine models, either when injected alone or in combination with S-NGR-TNF, a targeted inflammatory cytokine.

## Materials and methods

### Materials

Human serum albumin (Albutein, cat. A4AFC02342, Grifols); sulfosuccinimidyl 4-(N-maleimidomethyl)cyclohexane-1-carboxylate (sulfo-SMCC, Pierce, cat. A39268); gel-filtration NAP 5 and PD10 columns (Cytivia, cat. 17-0853-01 and cat. 17-0851-01); dialysis tubing (Spectra/Por^®^, cutoff 25 kDa; VWR, cat. 734 − 0504). Millex^®^GP syringe filter (Millipore, cat. SLGM33RS); phosphate-buffered saline (Merck, cat. P3813-1PAK, called *PBS-Sigma*). Precast 4–20% gradient polyacrylamide gel (Mini Protean TGX precast, BIO-RAD, cat. 456–1093). Mouse anti-human/mouse αvβ6 antibody (clone 10D5, IgG2a, Millipore, cat. MAB2077Z); rabbit anti-human/mouse β6 polyclonal antibody (Invitrogen, cat. PA5-35903); mouse anti-human/mouse αvβ8 antibody, clone ADWA-11 (a kind gift from Dr. Dean Sheppard); control isotype-matched murine IgG1, clone MOPC-31 C (Sigma, cat. M9035); rabbit anti-human αvβ8 monoclonal antibody, clone EM13309; rabbit anti-human/mouse β8 polyclonal antibody (Byobirt cat. orb184308), control isotype rabbit IgG (Abcam, cat. ab37415); Alexa Fluor 488-labeled goat anti-mouse (Invitrogen, cat. A-11001) and goat anti-rabbit (Invitrogen, cat. A-11034); Alexa Fluor 647-labeled goat anti-mouse (Invitrogen, cat. A-32728); human TGF-β (InvivoGen, cat. rcyc-htgfb1); HRP-labeled goat anti-rabbit polyclonal antibody (Sigma, cat. A4914); HRP-labeled streptavidin (Sigma, cat. S5512).

S-NGR-mTNF, a recombinant tumor necrosis factor-alpha (TNF) derivative, was prepared as described previously [21]. Recombinant human integrins were purchased from Bio-techne (αvβ1, cat. 6579-AVB-050; αvβ3, cat. 3050-AV-050; αvβ6, cat. 3817-AV-50; αvβ8, cat. 4135-AV-50 and α5β1, cat. 3230-A5-050). Biotinylated recombinant human αvβ6 and αvβ8 integrins were obtained from Acros CROBiosystems (cat. IT6-H82E4 and cat. IT8-H82W5, respectively).

### Cell lines

Human BxPC-3 pancreatic ductal adenocarcinoma (cat. CRL-168), human 5637 bladder carcinoma (cat. HTB-9), murine WEHI-164 fibrosarcoma (cat. CRL-1751), and murine TRAMP-C2 prostate cancer (cat. CRL-2731) cells were obtained from the American Type Culture Collection. Murine TS/A mammary adenocarcinoma cells were obtained from Sigma-Aldrich (cat. SCC177). Murine 5M7101 pancreatic cancer cells, which were isolated from spontaneous liver metastases of Ptf1a-Cre KrasG12D p53+/- KCP heterozygous mice [22], were a kind gift from Dr. Hana Algül (Technische Universität München, Germany). Murine K8484 PDAC cells, established from KPC mice (PdxCre/LSL-KrasG12D-Trp53R172H [23]), were a kind gift from Dr. Lorenzo Piemonti (San Raffaele Scientific Institute, Milan, Italy). 5M7101 and K8484 cells were engineered in-house to express green fluorescent protein (GFP) and human carcinoembryonic antigen (CEA) by lentiviral transduction (5M7101/GFP/CEA and K8484/GFP/CEA cells) as described previously [24]. Human T3M-4 pancreatic ductal adenocarcinoma cells were kindly gifted by Dr. Monica Casucci (San Raffaele Scientific Institute). BxPC-3, 5637, TS/A, and K8484/GFP/CEA cells were cultured in RPMI-1640 medium containing 10% heat-inactivated fetal bovine serum and standard supplements. WEHI-164, TRAMP-C2, 5M7101, and 5M7101/GFP/CEA cells were cultured in DMEM medium containing 10% heat-inactivated fetal bovine serum, and standard supplements with 1% nonessential amino acids added to 5M7101 and 5M7101/GFP/CEA. All the cell lines were free of mycoplasma, as routinely tested via the MycoAlert Control Set (Lonza).

### Preparation of induced regulatory T lymphocytes (iTregs) and conventional T lymphocytes

Regulatory T cells (iTregs, CD4^+^CD25^hi^CD127^low^ cells) and CD4^+^ conventional T cells (Tconvs, CD25^−^CD127^hi^CD45RA^+^ cells) were isolated from PBMCs by fluorescence-activated cell sorting, essentially as previously described [25], starting from buffy coats. For iTregs, sorted CD4^+^ and CD25^+^ T cells were stimulated with magnetic beads coated with an anti-CD3/anti-CD28 mAb (bead/T cells ratio, 3/1) and cultured in X-vivo medium, containing 10% human serum, 1% penicillin, 1% streptomycin, glutamine, and 100 nM rapamycin. After two days, the cells were supplemented with 500 U/ml IL-2. After 14 days, the activation beads were magnetically removed, and the cells were expanded in the presence of IL-2 (but without rapamycin) until day 21. On day 21, the phenotype was evaluated via cytometry. For Tconvs, sorted CD4^+^and CD25^−^ T cells were stimulated with anti-CD3/anti-CD28 magnetic beads (beads/T cell ratio, 3/1) and cultured in RPMI supplemented with 10% FBS, 1% penicillin, 1% streptomycin, 2 mM glutamine, 5 ng/ml IL-7, and 5 ng/ml IL-15. After 6 days, the activation beads were magnetically removed, and the cells were expanded until day 21 in the presence of IL-7 and IL-15.

### Preparation and characterization of peptides

Peptides were prepared by chemical synthesis, as described previusly [20, 26], dissolved in sterile water, and stored in aliquots at − 20 °C until use. The peptide concentration was determined by Ellman’s assay using 5,5-dithio-bis2-nitrobenzoic acid (DTNB, Ellman’s Reagent, Thermo Fisher, cat. 22582) and/or BCA assay (Thermo Fisher, cat. A55864). The identity and purity of the peptides were determined by mass spectrometry (MS) and reverse-phase high-performance liquid chromatography (HPLC) analysis.

### Conjugation of peptide 5a to IRDye fluorophores

Peptide **5a** (sequence: CFETLRGDLRILSILR**X**_**1**_QNL**X**_**2**_KELQD-amide, chemically stabilized by a triazole bridge between the propargylglycine (**X**_**1**_) and azidolysine (**X**_**2**_) residues) was coupled via its thiol group to IRDye^**®**^ 800CW maleimide or IRDye^**®**^ 680RD maleimide (LI-COR, P/N: 929-80020 and 929-71050, respectively), as described previously [26]. The resulting conjugates were called **5a**-IRDye800 and **5a**-IRDye680.

### Preparation and characterization of 5a-HSA

Human serum albumin (HSA) (2.5 ml, 500 mg) was gel-filtered through a PD10 column pre-equilibrated in 10 mM phosphate buffer, 138 mM sodium chloride, 2.7 mM potassium chloride, pH 7.4, containing 5 mM EDTA (*PBS-E*). The eluted product was sterilized by filtration, aliquoted, and stored at -80 °C until subsequent use. The product was then used to prepare the **5a**-HSA conjugate or a control conjugate lacking peptide **5a**, as follows: HSA (117.3 mg in 1.5 ml of *PBS-E*) was mixed with sulfo-SMCC (6.78 mg in 340 µl of water) and left to react for 1 h at room temperature (HSA: SMCC molar ratio, 1:9). The mixture was then purified by gel-filtration chromatography on a NAP-5 column (Cytiva) pre-equilibrated with *PBS-E*. The eluted product, corresponding to activated-HSA, was then diluted to 4.5 ml with *PBS-E* and divided into two separate tubes (2.25 ml/each). To one tube, we added 4.80 µmol of peptide **5a** in 0.45 ml aliquots every 20 min (final peptide/HSA molar ratio of ∼6:1), while 2-mercaptoethanol (negative control, lacking the peptide) was added to the other tube. The mixtures were left to react overnight at 4 °C and quenched with 2-mercaptoethanol (1 mM, final concentration) for 0.5 h. The resulting products (5 ml final volume in *PBS-Sigma*) were gel-filtered using two PD10 columns in parallel pre-equilibrated with *PBS-Sigma*. The products were pooled (7 ml final volume) and dialyzed (cutoff: 25 kDa) overnight against 50 mM sodium phosphate, pH 7.4, containing 150 mM sodium chloride. The products were then aliquoted and stored at − 20 °C. The protein concentration of each conjugate (**5a**-HSA and *HSA) was determined spectrophotometrically (A_280_ nm using an adsorption coefficient of 0.54). The identity and purity of the final product were characterized by (a) SDS-PAGE using 4–20% precast polyacrylamide gels, (b) MALDI-TOF mass spectrometry analysis, and (c) gel-filtration chromatography using a Superdex 75 h column (Cytivia) connected to an HPLC instrument (AKTA purifier 10).

### MALDI-TOF mass spectrometry analysis

The peptide-albumin conjugates were diluted in deionized water (100–300 µg/ml) and 1 µl of the solution was spotted onto an MTP 384 ground steel target plate (Bruker Daltonics). A saturated α-cyano-4-hydroxycinnamic acid solution, prepared in 50:50 acetonitrile: water with 0.1% TFA, was spotted (1 µl) onto the sample using the double-layer method. Mass spectrometry analyses were conducted using a Bruker rapifleX™ MALDI TissueTyper™ MALDI-TOF/TOF operating in linear positive mode within the 20–200 kDa mass range and employing the M5-Thin layer laser setting. External calibration was performed using bovine serum albumin. The spectra were exported to flexAnalysis Version 4.2 (Build 14; Bruker Daltonics) for baseline removal, smoothing, and manual feature selection.

### Integrin binding assays

The ability of **5a**-HSA to recognize αvβ6 and αvβ8 integrins was investigated using (a) direct binding assay based on the use of integrin-coated plates in the capture step and an anti-HSA polyclonal antibody in the detection step (*Assay 1*), (b) competitive assays based on the use of integrin-coated plates and isoDGR-HRP conjugate as a probe for the RGD-binding site of integrins (*Assay 2*), or (c) competitive assays based on the use of latent TGFβ1-coated plates and biotinylated αvβ6 or αvβ8 followed by HRP-labeled streptavidin (*Assay 3*).

#### Direct integrin binding assay (Assay 1)

Ninety-six-well PVC microtiter plates (Carlo Erba, cat. FA5280100) were coated with or without human recombinant αvβ6 and αvβ8 integrins in Dulbecco’s phosphate-buffered saline containing calcium and magnesium (DPBS) (1 µg/ml, 50 µl/well, overnight at 4 °C). After washing, the plates were blocked with 3% BSA in DPBS (150 µl/well) and incubated for 1 h at room temperature. The plates were then washed with 25 mM Tris–HCl buffer, pH 7.4, containing 150 mM sodium chloride, 1 mM magnesium chloride, 1 mM manganese chloride, and 0.05% Tween-20 (*Buffer-1*), and filled with various amounts of **5a**-HSA (50 µl/well) in *Buffer-1* containing 1% w/v BSA and 0.05% Tween-20 (*Buffer-2*). After 1.5 h of incubation, the plates were washed with *Buffer-1* and filled with an anti-human HSA antibody (Sigma, cat. A3293, 1:10000, 50 µl/well, in *Buffer-2* containing 1% normal goat serum (NGS) and 0.05% Tween-20 (*Buffer-3*)). After 1 h of incubation, the plates were washed and filled with HRP-labeled goat anti-rabbit polyclonal antibody (1:1000, 50 µl/well, 1 h, *Buffer-3*). After washing, bound peroxidase was detected by adding o-phenylenediamine, a chromogenic substrate, and the absorbance was measured at 490 nm. The specific binding of **5a**-HSA was determined by subtracting the nonspecific binding measured in the wells coated without integrin. The affinity constant (*Kd*) was then calculated using the “*one-site specific binding*” equation in Prism software.

#### Competitive integrin binding assays (Assay 2 and 3)

The competitive integrin binding *assay two* was performed as previously described [20]. Briefly, various amounts of **5a**-HSA were mixed with a fixed amount of the isoDGR-HRP conjugate and added to integrin-coated plates. After two hours of incubation, the plates were washed, and bound peroxidase was detected, as described above. The inhibitory constant (*Ki*) was calculated as previously described [20].

The competitive integrin binding *assay 3* was performed as follows: ninety-six-well microtiter plates (Greiner Bio-One, cat. #675061) were coated with or without human recombinant latent TGFβ1 (Acro Biosystems, cat. TG1-H524x) in 50 mM sodium carbonate buffer, pH 9.5, containing 1 mM calcium chloride, 1 mM manganese chloride (2 µg/ml, 50 µl/well overnight at 4 °C). After washing with 20 mM Tris-HCl pH 7.4, containing 150 mM sodium chloride, 1 mM manganese chloride, and 0.05% Tween-20 (*Buffer-4*), the plates were blocked with 2% BSA in *Buffer-4* (150 µl/well) and incubated for 1.5 h at 37 °C. The plates were then washed with *Buffer-4* and filled with various amounts of peptide **5a** or **5a**-HSA and 10 ng/ml biotinylated recombinant human αvβ6 or αvβ8 integrins (50 µl/well) in *Buffer-4* containing 0.5% w/v BSA (*Buffer-5*). After 1 h of incubation, the plates were washed with *Buffer-4* and then filled with HRP-labeled streptavidin (1:2000 in *Buffer-5*, 50 µl/well, 1 h). After washing, the bound peroxidase was detected by adding the chromogenic substrate 3,3′,5,5′-tetramethylbenzidineo-phenylenediamine (Sigma, cat. T3405, prepared according to the manufacturer’s instructions), and after 10 min, the absorbance was measured at 450 nm.

### Cell adhesion assays

Cell adhesion assays were carried out as described previously [27], except that the cells were seeded into 96-well plates with their own cell culture medium containing 3% BSA.

### Flow cytometry analysis

Flow cytometry analysis of cell surface αvβ6 and αvβ8 expression was carried out as described previously [20, 26]. Binding assays of **5a**-IRDye680 and **Cys**-IRDye680 to cells were carried out by incubating trypsin-EDTA detached cells with various amounts of conjugates (ranging from 0 to 200 nM) in 25 mM HEPES buffer, pH 7.4, containing 150 mM sodium chloride, 1 mM magnesium chloride, 1 mM manganese chloride, and 2% w/v BSA (1 h on ice). After washing, the cells were fixed with 2% paraformaldehyde in PBS, and bound fluorescence was detected using a CytoFLEX S flow cytometer (Beckman Coulter) equipped with a 638 nm laser and 712/25 nm band-pass filter set. Flow cytometry data were analyzed using the FlowJo software (BD Biosciences).

### In vivo studies in animal models

The anti-tumor properties of **5a**-HSA were assessed using mouse models of pancreatic cancer liver metastasis and subcutaneous mouse tumor models of fibrosarcoma, mammary adenocarcinoma, and prostate cancer.

#### Mouse models of pancreatic cancer liver metastasis

5M7101/GFP/CEA or K8484/GFP/CEA pancreatic ductal adenocarcinoma cells were implanted into the liver of 8-10-week-old C57BL/6 N female and male mice, respectively. For this purpose, 1.0 × 10^5^ cells in 200 µl of phosphate-buffered saline were injected into the portal vein, as previously described [28, 29]. After 2–3 weeks, tumor growth was monitored using a 7-Tesla magnetic resonance imaging (MRI) system (BioSpec, Bruker BioSpin Gmbh) equipped with 450/675 mT/m gradients (slew rate: 3400–4500 T/m per second; rise time: 140 µs) coupled with a circular polarized mouse body volume coil (inner diameter of 40 mm) using gadoxetic acid as a contrast agent (Gd-EOB-DTPA; Primovist, Bayer Schering Pharma, administered via the tail vein before imaging, 0.05 µmol/g of body weight). The MRI protocol included axial fat-saturated T2 weighted sequences acquired across the entire abdomen (TurboRARE-T2: TR = 3080 ms, TE = 40 ms; voxel-size, 0.161 × 0.116 × 0.8 mm; average = 5), followed by an axial fat-saturated T1-weighted sequence (RARE-T1: TR = 500 ms, TE = 7.6 ms, voxel-size = 0.161 × 0.116 × 0.8 mm, average = 5) with the same field of view acquired after Gd-EOB-DTPA injection, during the hepatobiliary phase of contrast excretion (starting from 10 min after Gd-EOB-DTPA injection). Normal liver tissue appears hypointense on T2-weighted images and hyperintense on hepatobiliary phase T1-weighted images. Liver metastases were defined as focal lesions characterized by slight hyperintensity on T2-weighted images and concurrent hypointensity on hepatobiliary phase T1-weighted images. The tumor volume and number of metastases were quantified by a certified radiologist with > 4 years of experience in preclinical abdominal MRI (blinded to any other information) using the open-source Medical Imaging Processing, Analysis, and Visualization software (MIPAV, version 11.0.7, Biomedical Imaging Research Services Section, ISL, CIT, National Institute of Health, USA). Regions-of-interest (ROIs) were drawn slice-by-slice on liver metastases, and the volume of interest (VOI) of each lesion was calculated by multiplying the ROIs areas (mm^2^) by the slice thickness (mm). Finally, the total metastatic volume was obtained by summing the volumes of all the single VOIs. The animals were sacrificed before the tumors reached a diameter of 1–1.5 cm.

#### Subcutaneous mouse tumor models

BALB/c or C57BL/6 N mice (Charles River Laboratories) were challenged with a subcutaneous injection in the left flank of 1.5 × 10^6^ WEHI-164 fibrosarcoma cells or 3 × 10^5^ TS/A cells (BALB/c female mice, 18–20 g) or 2.5 × 10^6^ TRAMP-C2 prostate cancer cells (C57BL/6 N male mice, 7–8 weeks-old). Tumor growth was monitored by measuring the tumor size using calipers. Tumor volume was estimated by calculating r1×r2×r3 × 4/3π, where r1 and r2 are the longitudinal and lateral radii, respectively, and r3 is the thickness of the tumor protruding from the surface of the normal skin. The animals were sacrificed before the tumors reached a diameter of 1–1.5 cm. Tumor sizes are shown as the mean ± SE.

#### In vivo treatments

Mice were injected i.p. with **5a**-HSA alone (40–50 µg/mouse diluted in 0.9% sodium chloride solution containing 100 µg/mL HSA) or in combination with S-NGR-TNF (100 pg, 5 ng/kg, in 0.9% sodium chloride solution containing 100 µg/mL HSA) as indicated in each corresponding figure.

### TGFβ bioassay

The quantification of bioactive TGFβ in cell supernatants was carried out using TGFβ-Reporter HEK-Blue™ cells (InvivoGen) according to the supplier’s recommendations.

### Detection of phospho-SMAD2/3 in tumors by Western blot and immunohistochemical analyses

#### Western blot analysis of phospho-SMAD3 in tumor extracts

The effect of **5a**-HSA on TGFβ activation in tumors in in vivo animal models was assessed by western blot analysis of phospho-SMAD3, a critical intracellular mediator of TGFβ signaling, in tumor tissue extracts. Tumors were excised from euthanized mice after treatment with or without **5a**-HSA and homogenized in 50 mM Tris-HCl, pH 8.0, 150 mM sodium chloride, 0.1% (w/v) sodium dodecyl sulfate, 0.5% (w/v) sodium deoxycholate, and 1% (v/v) NP-40 (1 ml/g tissue) containing a cocktail of protease and phosphatase inhibitors (Abcam, cat. #ab201119). The protein concentration of the lysate was measured using the BCA™ Protein Assay Kit (Thermo Fisher Scientific cat. #23227). Tumor lysates containing 100–350 µg of total protein were (a) diluted 1:1 in 2x Laemmli sample buffer (Bio-Rad, cat. #1610737) supplemented with 10% v/v 2-mercaptoethanol, (b) boiled at 95 °C on a heat block, (c) separated by SDS-PAGE on a 10% polyacrylamide gel (Mini-PROTEAN^®^ TGX™ Precast, Bio-Rad), and transferred to a polyvinylidene difluoride membrane (Transfer Packs, Bio-Rad cat. #170415) using a Trans-Blot Turbo Mini device (Bio-Rad). The membranes were then soaked in 20 mM Tris-HCl, pH 8.0, 150 mM sodium chloride, 0.1% Tween 20 (TBS-T) containing 5% (w/v) bovine serum albumin (BSA) (*Blocking Buffer*) for 1 h at room temperature. After washing with TBS-T, the membranes were incubated overnight at 4 °C with the following primary antibodies in *Blocking Buffer*: a rabbit anti-phospho-SMAD3 (S423 and S425) mAb (EP823Y clone, Abcam, cat. #ab52903, 1:1000) or a rabbit anti-SMAD3 mAb (EP568Y clone, Abcam cat. #ab40854, 1:30000). Both antibodies were mixed with a rabbit polyclonal anti-actin antibody (Sigma cat. #A2066, 1:5000-1:20000). The membranes were then washed five times with TBS-T (3 min each) and incubated with a horseradish peroxidase (HRP)-goat anti-rabbit polyclonal antibody conjugate (Sigma cat. #A4914, 1:40000, 1 h at room temperature). After additional washing, the membranes were incubated with a chemiluminescent HRP substrate (Amersham, cat. #RPN2232). Antibody binding was detected using a ChemiDoc Imaging System (Bio-Rad), and band densitometric analysis was performed using the ImageJ software.

#### Immunohistochemical analysis of phospho-SMAD2/3 in tumor tissue sections

The livers of mice with PDAC metastases were explanted from euthanized mice and fixed in zinc formalin solution for 24 h at room temperature. The livers were subsequently embedded in paraffin and processed according to the standard immunohistochemical procedures at the San Raffaele Mouse Clinic. Phospho-SMAD2/3 staining of the tumor sections was conducted as previously described, with minor modifications [30]. The tissue sections were rehydrated at room temperature, followed by antigen retrieval using 10 mM sodium citrate buffer, pH 6.0 (Sigma-Aldrich, cat. S1804). Endogenous peroxidase activity was then blocked by treating the sections with methanol containing 0.03% hydrogen peroxide for 10 min at room temperature. Subsequently, the sections were incubated for 30 min at room temperature with a rabbit anti-phospho-SMAD2/3 polyclonal antibody (Santa Cruz Biotechnology, cat. SC-11769) (5 µg/ml) After washing, the sections were incubated with a biotinylated goat anti-rabbit polyclonal antibody conjugate (Dako, cat. E0432) (1:500, 1 h at room temperature), followed by an avidin-biotin-peroxidase complex according to the manufacturer’s instructions (Vector Lab). Antibody binding was detected using 3,3′-diaminobenzidine (DAB; Dako) staining. The tissue slices were imaged using an Aperio Digital Pathology Slide Scanner (Leica Biosystems). The brown signal area, corresponding to the reaction product precipitated into the cell nuclei, was quantified using QuPath software [31] as follows: small lesions were quantified by drawing a single ROI covering the entire liver metastasis, and large lesions were quantified by drawing multiple ROIs randomly selected within each lesion (> 3 ROIs per lesion).

## Results

### Preparation and characterization of 5a-HSA

**Fig. 1:**
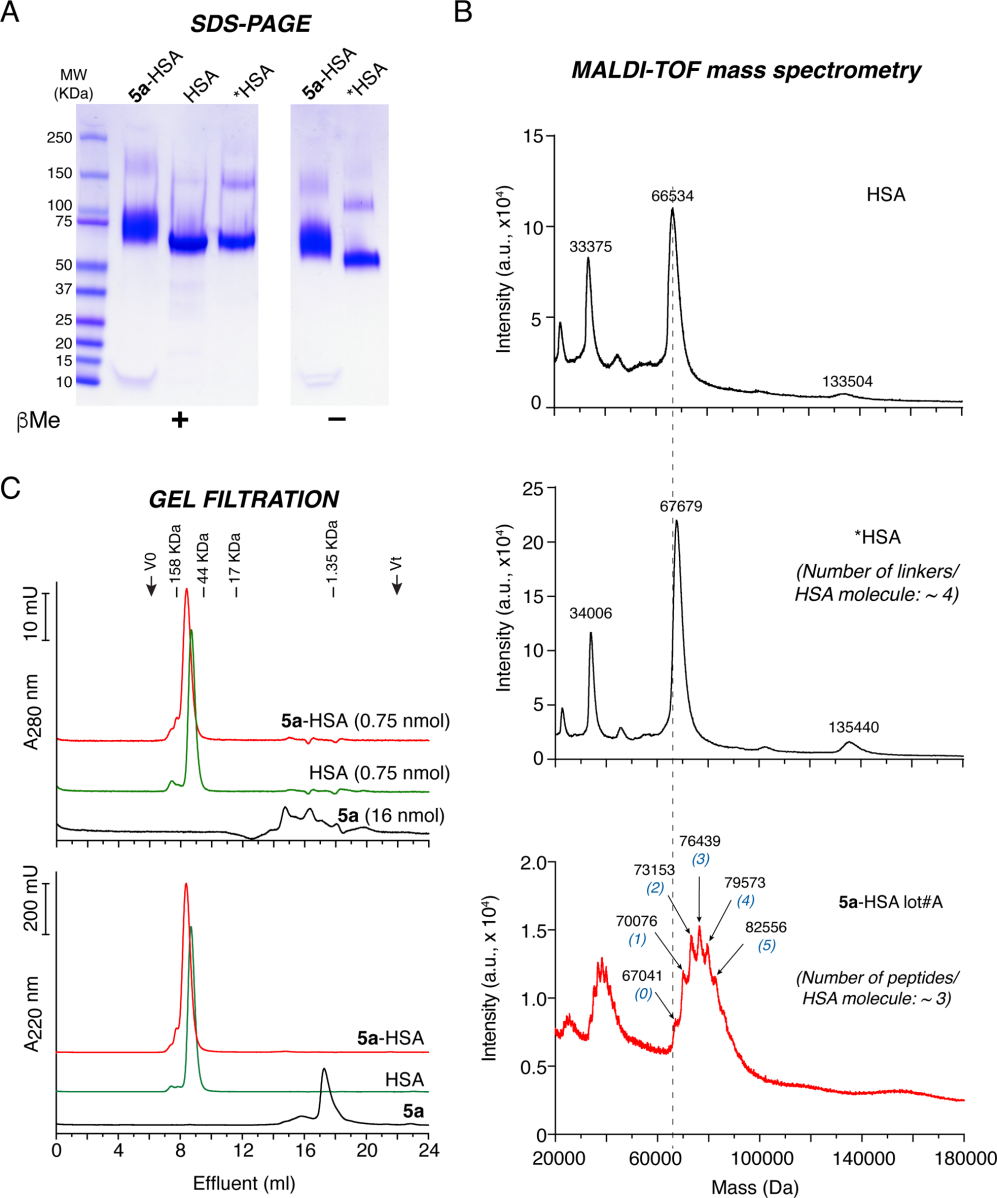
Biochemical characterization of **5a**-HSA (Lot #A). **A**) SDS-PAGE analysis of **5a**-HSA, HSA, and linker-HSA (*HSA) under reducing (+βMe) and non-reducing conditions (−βMe). **B**) MALDI-TOF mass spectra of HSA, *HSA, and **5a**-HSA (lot #A). The average masses of the principal components and the estimated number of peptides coupled to HSA molecules are indicated. **C**) Analytical gel-filtration chromatography of **5a**-HSA, HSA, and peptide **5a** on a Superdex 75 h column. Void volume (*Vo*), total volume (*Vt*), and elution volume of molecular markers (158, 44, 17, and 1.35 kDa) are indicated

Peptide **5a** was chemically coupled to human serum albumin (HSA) using the hetero-bifunctional sulfosuccinimidyl 4-(N-maleimidomethyl)cyclohexane-1-carboxylate (sulfo-SMCC) cross-linking agent (**5a**-HSA). To assess the number of peptide moieties/HSA molecule in the final product, we analyzed the molecular weights of HSA, sulfo-SMCC-activated HSA (*HSA), and **5a**-HSA (lot #A) using SDS-PAGE under reducing and non-reducing conditions, mass spectrometry, and gel-filtration chromatography. The results showed that this conjugate consists of a mixture of monomeric molecules with molecular weights ranging from ∼70 to ∼90 kDa (Fig. 1A-C). Considering that the molecular weights of HSA and peptide **5a** plus linker are ∼66.5 and ∼3.33 kDa, respectively, we estimate from these data that different HSA molecules bearing different numbers of peptides (ranging from 1 to 6, average ∼3) were present in the final product. Similar results were obtained by mass spectrometry analysis of a second preparation of **5a**-HSA (lot #B), again showing a heterogeneous conjugate with an average of ∼4 coupled peptides/HSA molecule (Supplemental Fig. S1).

### 5a-HSA binds Recombinant human αvβ6 and αvβ8

**Fig. 2:**
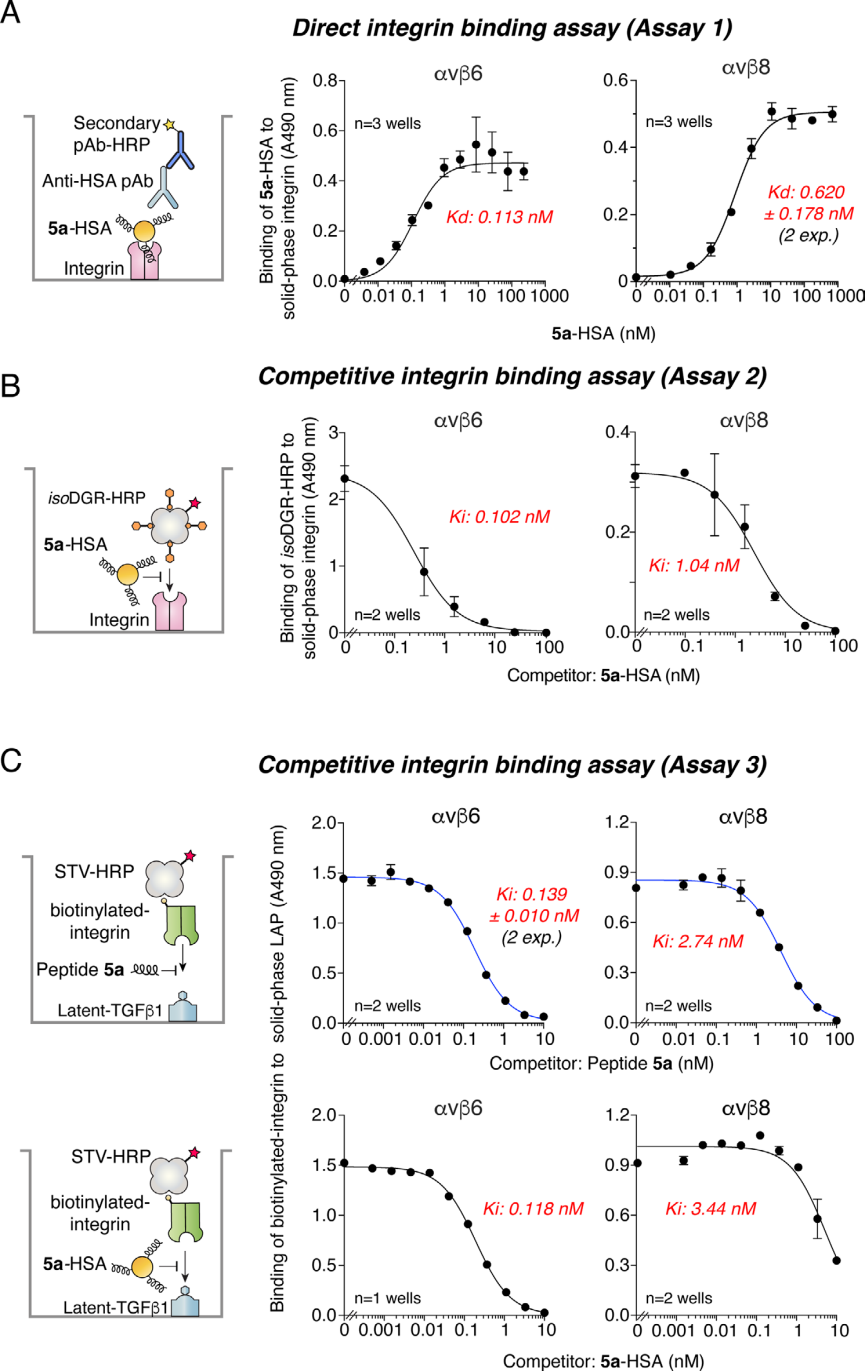
Binding of **5a**-HSA and peptide 5a to recombinant human αvβ6 and αvβ8 (competitive and direct binding assays). Schematic representation of the assays (*left panels*) and dose-response curves (*middle-right panels*). **A)** Direct binding of **5a**-HSA to αvβ6- or αvβ8-coated microtiter plates as detected with an anti-HSA rabbit polyclonal antibody and HRP-labeled goat anti-rabbit antibody. **B**) Competitive binding of **5a**-HSA and *iso*DGR-HRP conjugate to αvβ6- or αvβ8-coated microtiter plates. **C**) Competitive binding of peptide **5a** (***upper panels***) or **5a**-HSA (***lower panels***) and biotinylated-αvβ6 or -αvβ8 integrins to latent TGFβ1-coated microtiter plates, as detected with HRP-labeled streptavidin (STV-HRP). Dots, mean ± SE of technical duplicates. Inhibition constant (*Ki*) or dissociation constant (*Kd*) are also indicated in each panel

The binding properties of **5a**-HSA to purified human integrin αvβ6 and αvβ8 were investigated using direct and competitive binding assays (see *Methods* and Fig. 2 for schematic representations of the assays).

Direct integrin-binding assays, based on the use of microtiter plates coated with purified human integrins αvβ6 or αvβ8, showed that **5a**-HSA could bind both integrins with high affinity (*Kd* values of 0.113 nM and 0.620 nM, respectively) (Fig. 2A, Supplemental Table S1). Similar results were obtained using competitive binding assays based on the use of the isoDGR-HRP conjugate as a probe for the RGD-binding site of these integrins (Fig. 2B). Other competitive binding assays, based on the use of the latent TGFβ1 in the solid-phase and biotinylated αvβ6 or αvβ8 in the liquid phase, showed that peptide **5a** and **5a**-HSA could compete for the interaction between latent TGFβ1 and αvβ6 or αvβ8 integrin with similar or even better potency than peptide **5a** (Fig. 2C, Supplemental Table S1). Overall, these results indicate that coupling peptide **5a** to HSA preserves its ability to recognize αvβ6 and αvβ8.

### 5a-HSA recognizes αvβ6 and/or αvβ8-positive cancer cells

The ability of **5a-**HSA to recognize αvβ6^pos^ and/or αvβ8^pos^ cancer cells was investigated. To this end, we first characterized the expression of αvβ6 and αvβ8 in murine cells, such as TS/A mammary adenocarcinoma, WEHI-164 fibrosarcoma, TRAMP-C2 prostate adenocarcinoma, 5M7101/GFP/CEA, and K8484/GFP/CEA pancreatic ductal adenocarcinoma, as well as in human cells, such as BxPC-3 pancreatic ductal adenocarcinoma and 5637 bladder cancer. Integrin expression in these cells was assessed by FACS using anti-αvβ6 and -αvβ8 antibodies. The results showed that the 5637 cell line expresses both integrins (αvβ6^pos^, αvβ8^pos^), whereas BxPC-3 cells express only αvβ6 (αvβ6^pos^, αvβ8^neg^), and TRAMP-C2 cells only αvβ8 (αvβ6^neg^, αvβ8^pos^). At variance, 5M7101/GFP/CEA and K8484/GFP/CEA showed low-to-moderate expression of αvβ6 and no detectable αvβ8, while TS/A and WEHI-164 cells showed no expression of αvβ6 or little expression of αvβ8 (Supplemental Fig. S2). Based on these integrin expression patterns, the 5637, BxPC-3, and TRAMP-C2 cell lines were selected for **5a-**HSA binding studies.

Cell adhesion assays showed that **5a**-HSA, but not *HSA, promoted the adhesion of both single- and double-positive cells to microtiter plates coated with these proteins (Supplemental Fig. S3A). Notably, the adhesion of TRAMP-C2 cells to **5a-**HSA-coated plates was completely blocked by an excess of peptide **5a** added to the cells but not by peptide **2a** (a control peptide containing the RGE sequence in place of RGD) (Supplemental Fig. S3B). Overall, these results suggest that **5a-**HSA interacts with αvβ6 and/or αvβ8 when expressed on the cell membrane.

### Peptide 5a and 5a-HSA inhibit the production of active TGFβ by cancer cells

**Fig. 3:**
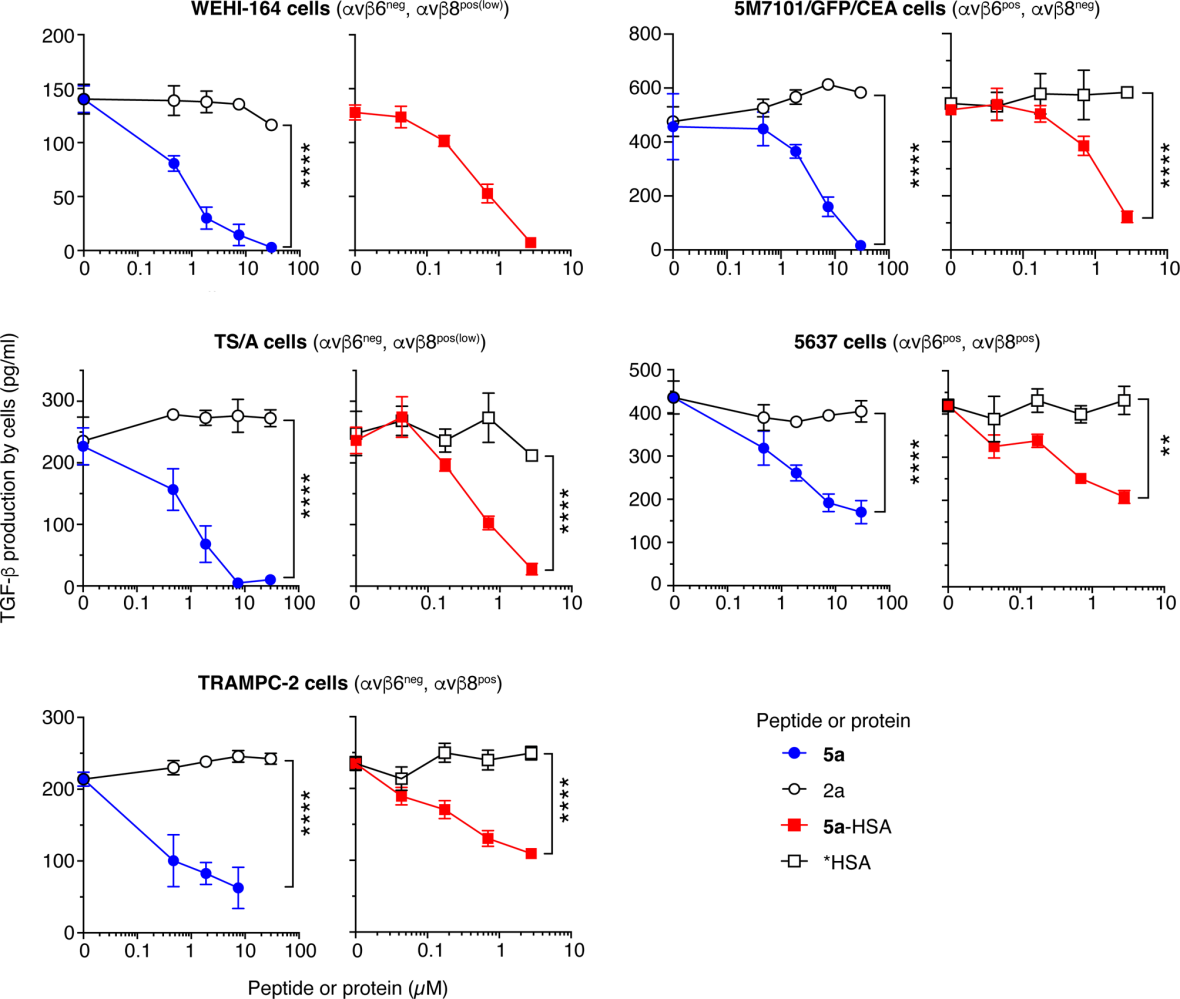
Peptide **5a** and **5a**-HSA inhibit the production of active TGFβ by cancer cells. Effect of **5a**, **2a**, *HSA, and **5a**-HSA on TGFβ production by WEHI-164, TS/A, TRAMP-C2, 5M7101/GFP/CEA, and 5637 cells. The cells were seeded on cell culture microplates, allowed to adhere for 2 h, and treated with the indicated compounds for 48–72 h. The amount of active TGFβ in the supernatant was quantified using a bioassay based on HEK-Blue™ TGFβ cells (see Methods). The results of one representative experiment per each cell line are shown (mean ± SE, *n* = 2–4 wells). **, *P* < 0.01; ****P < 0.0001 by two-tailed t-test

To verify that **5a**-HSA can efficiently inhibit TGFβ activation mediated by αvβ6- or αvβ8-positive cancer cells, we investigated the effect of **5a**-HSA, *HSA (a negative control protein), and peptide **5a** and **2a** (a negative control peptide) on TGFβ production by WEHI-164, TS/A, TRAMP-C2, 5M7101/GFP/CEA, and 5637 cancer cells. The amount of active TGFβ in the cell supernatant was quantified after 48–72 h of incubation using a bioassay based on HEK-Blue™ TGFβ cells. As expected, **5a** and **5a**-HSA, but not **2a** and *HSA, inhibited the production of TGFβ in a dose-dependent manner (Fig. 3), presumably by inhibiting integrin-mediated activation of its latent form.

### Peptide 5a inhibits Treg-mediated TGFβ activation

**Fig. 4:**
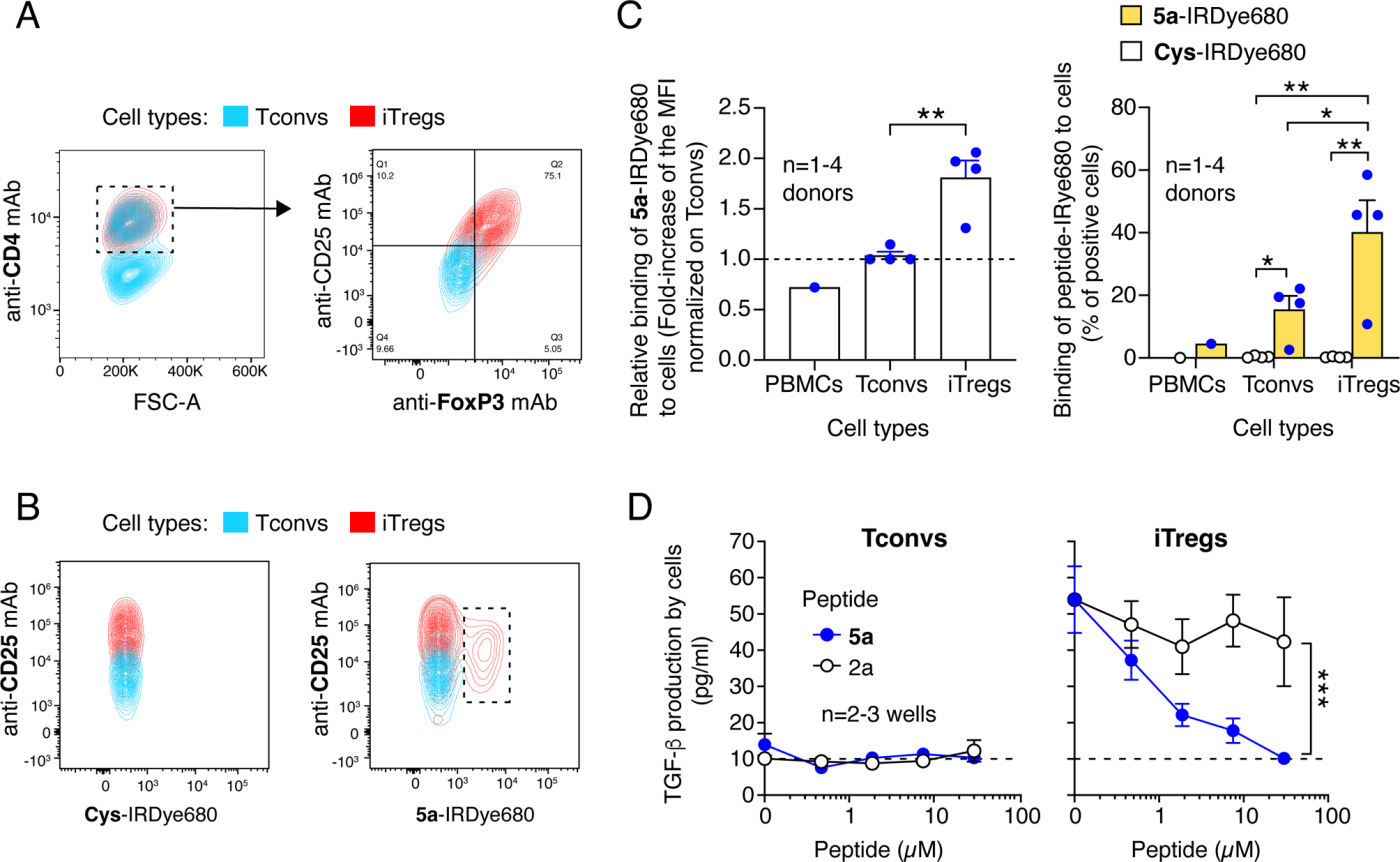
Peptide **5a** binds to human iTregs and inhibits the production of active TGFβ by iTregs. **A**) Characterization of conventional T cells (Tconvs) and induced T-regulatory cells (iTregs) by FACS. Representative overlaid two-parameter FACS plots of Tconvs (*blue contour plot*) and iTregs (*red contour plot*) populations stained with the indicated antibodies. The gating strategy used to identify the cell subset is shown (dashed rectangle with the arrow). **B-C**) Binding of **5a**-IRDye680 and **Cys**-IRDye680 to peripheral blood mononuclear cells (PBMC), Tconvs and iTregs. **B**) Representative overlaid two-parameter FACS plots of Tconvs (*blue contour plot*) and iTregs (*red contour plot*) incubated with **Cys**-IRDye680 or **5a**-IRDye680 (200 nM) in 25 mM HEPES buffer, pH 7.4, containing 150 mM sodium chloride, 1 mM magnesium chloride, 1 mM manganese chloride and 2% w/v BSA for 1 h. The dashed rectangle delineates a subset of iTregs, which could bind **5a**-IRDye680. **C**) Binding of **5a**-IRDye680 and **Cys**-IRDye680 to PBMCs, Tconvs or iTregs as determined by FACS. Specific binding is depicted as the ratio of the mean fluorescence intensity of **5a**-IRDye680 over **Cys**-IRDye680 (*left panel*) and the percentage of IRDye680 positive cells (*right panel*). Cumulative results of 1–3 experiments are shown, performed with cells isolated from 1–4 donors (*dots*), Bar, mean + SE. *, *P* < 0.05; **, *P* < 0.01; by two-way ANOVA with post-hoc Tukey’s multiple comparisons test. **D**) Effect of peptides **5a** and **2a** on active TGFβ production by Tconvs and iTregs. Cells were cultured for 25 days before the assay; on the day of the assay, they were seeded in a 96-well microplate and treated with the indicated peptides for 48 h. The amount of active TGFβ in the supernatant was quantified using a bioassay based on HEK-Blue™ TGFβ cells. The results of one experiment are shown (mean ± SE, *n* = 2–4 wells). The dotted line indicates the lower limit of detection of the assay. ***, *P* < 0.001 by two-way ANOVA with post-hoc Tukey’s multiple comparisons test

Given that regulatory T cells (Tregs) in the tumor microenvironment express αvβ8 and may activate TGFβ via this integrin [18, 32, 33], we hypothesized that peptide **5a** could bind these cells and inhibit their TGFβ-activating function. Significant binding of fluorescence-labeled peptide **5a** (**5a**-IRDye680) to in vitro differentiated regulatory T cells (iTregs) and little or no binding to PBMCs or conventional human T cells (Tconvs) was observed by FACS analysis of cells after incubation with this conjugate (Fig. 4A-C). Peptide **5a**, but not **2a**, blocked TGFβ activation in iTreg cultures (Fig. 4D). No TGFβ activation and no effect of peptides were observed with Tconvs (Fig. 4D). Notably, although no binding of an anti-human αvβ8 mAb (clone EM13309) to iTregs was observed by FACS analysis, mRNA analysis of these cells revealed the production of mRNA encoding β8 integrin (data not shown).

These results indicate that iTreg cells may represent another important target for peptide **5a** and its derivatives.

### 5a-HSA has a plasma half-life longer than that of peptide 5a

The plasma half-life of the fluorescence-labeled peptide **5a** injected i.p., is very short (~ 8 min) (Supplemental Table S2). To assess whether the conjugation of this peptide to HSA increases its plasma half-life, we administered **5a**-HSA (50 µg, i.p.) to mice and analyzed its plasma levels at various time points using ELISA. The plasma half-life of intraperitoneally injected **5a**-HSA was 15–17 h, as estimated from the curve obtained. Notably, 2-2.5 days after injection, the circulating levels of **5a**-HSA were still above the *Kd* values for both αvβ6 and αvβ8 integrins (Supplemental Fig. S4). The plasma half-life of the intravenously injected **5a**-HSA was 100 min (Supplemental Fig. S4).

### 5a-HSA inhibits TGFβ-signaling in αvβ6- or αvβ8-positive tumors

**Fig. 5:**
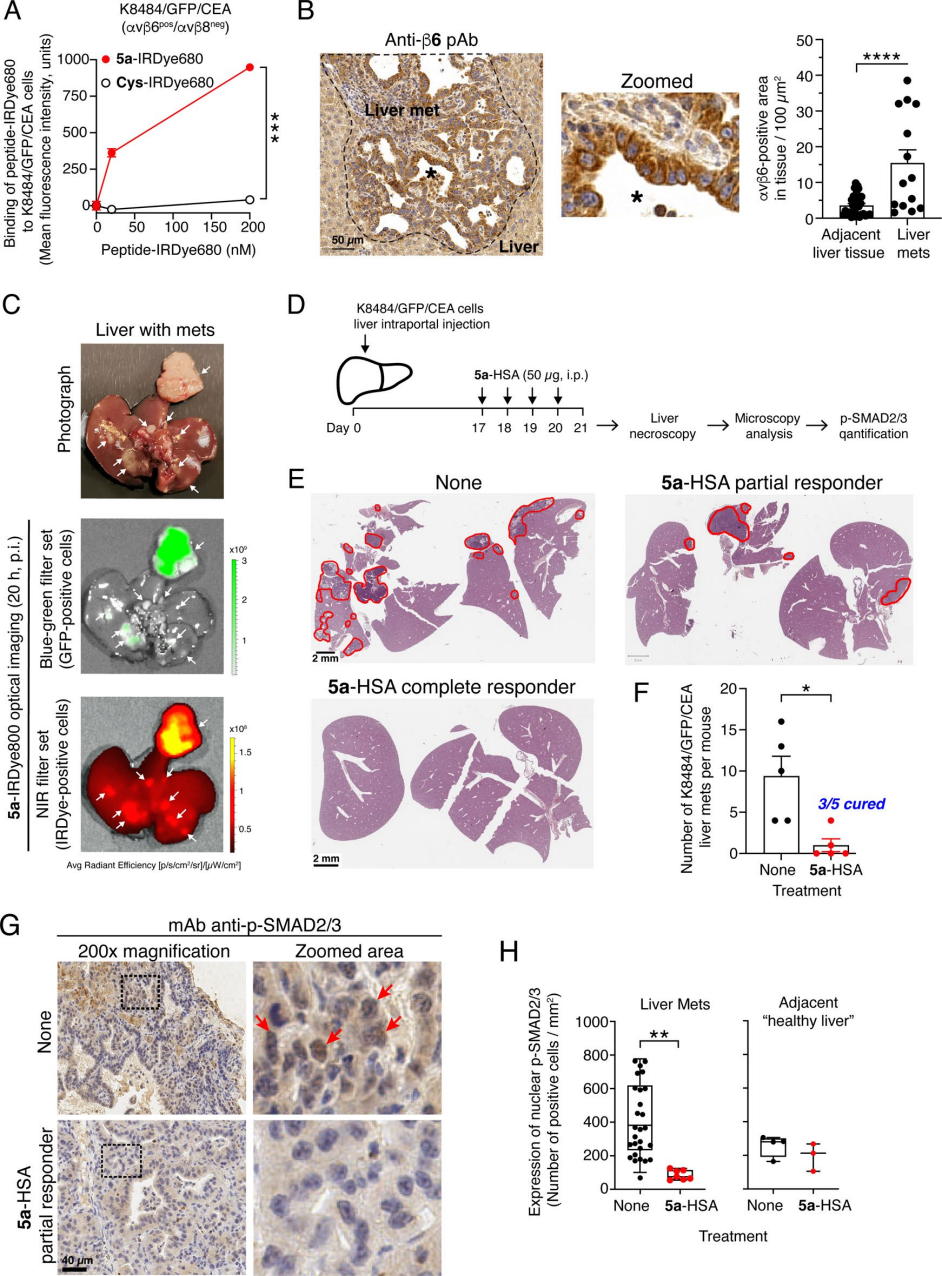
**5a**-HSA inhibits TGFβ signaling in K8484/GFP/CEA liver metastases. **A-C**) Characterization of K8484/GFP/CEA liver metastases. **A**) Binding of **5a**-IRDye680 and **Cys**-IRDye680 to K8484/GFP/CEA cells, as detected by flow cytometry. *Dots*, mean ± SD of technical duplicates. ***, *P* < 0.001 by unpaired two-tailed *t-test* analysis of the area under the curve for each peptide-IRDye680 binding curve calculated with GraphPad Prism software. **B**) Expression of integrin αvβ6 in mouse livers with K8484/GFP/CEA metastases. C57BL/6 N male mice were injected with 1.0 × 10^5^ K8484/GFP/CEA cells into the portal vein, and liver tumor colonies were left to grow. On day 32, the liver was excised, embedded in paraffin, and processed for immunostaining with a rabbit anti-β6 polyclonal antibody, followed by incubation with a secondary HRP-labeled goat anti-rabbit polyclonal antibody and DAB substrate. A representative image of liver tissue with metastases (outlined by the dashed line) and quantification of αvβ6 expression are shown (*n* = 4 mice/group; >3 mets per liver were quantified). ****, *P* < 0.0001 by unpaired two-tailed *t-test* analysis. **C**) Representative ex vivo images of the liver with K8484/GFP/CEA metastases in mice injected with 1.2 pmol of **5a**-IRDye800. *Arrows*, liver metastases; *green signal*, GFP-positive cells; *red signal*, **5a**-IRDye800-positive cells. **D-H)** Effect of **5a**-HSA on tumor growth and TGFβ signaling in mice bearing K8484/GFP/CEA liver mets, as detected by microscopy analysis and immunohistochemical quantification of p-SMAD2/3 **D**) Experimental scheme. K8484/GFP/CEA cells were injected into the portal vein as described above. Seventeen days later, the mice were randomized into two experimental groups: one group (*n* = 5) received **5a**-HSA, while the other group (*n* = 5) remained untreated (*None*). On day 21, the mice were sacrificed, and their livers were explanted for necroscopic and microscopic examination, followed by quantification of p-SMAD2/3. **E)** Representative microphotographs of liver tissue sections counterstained with hematoxylin and eosin from untreated and **5a**-HSA-treated mice. *Red lines* indicate liver metastases. **F**) Quantification of the number of PDAC liver mets in tissue sections. Bars represent the mean ± SE (*n* = 5 mice per group). Note that three out of five mice treated with **5a**-HSA showed no countable liver metastases (i.e., a complete response, *CR*), as assessed by visual inspection of the tissue sections by bright-field microscopy. **(G)** Immunohistochemical analysis of p-SMAD2/3 expression in mice bearing K8484/GFP/CEA liver mets treated with or without **5a**-HSA. Representative microphotographs (acquired at 200x magnification and electronically zoomed-in areas delineated by *black dashed rectangles*) of p-SMAD2/3 staining of liver metastases in untreated or **5a**-HSA-treated mice are shown. *Red arrows* indicate positive nuclei. **(H)** Quantification of nuclear p-SMAD2/3 positive cells in liver metastases and adjacent healthy livers. The number of positive nuclei was quantified using QuPath software and is shown as box-plots with 5–95 percentile, median, and min-to-max values (*n* = 2–5 mice per group, ≥1 mets per liver were quantified). *, *P* < 0.05, **P < 0.01 by unpaired two-tailed *t-test* analysis

To assess whether circulating **5a**-HSA can reduce TGFβ activation in the tumor microenvironment, we analyzed the activation of intracellular TGFβ signaling mediators (SMAD2/3) in different models of tumor-bearing mice after systemic administration of **5a**-HSA. We first investigated a model of pancreatic ductal adenocarcinoma (PDAC) hepatic metastases, which involves intraportal injection of murine K8484 PDAC cells genetically engineered to express green fluorescent protein (GFP) and carcinoembryonic antigen (CEA) (K8484/GFP/CEA), into immunocompetent mice. This approach allows tumor cells to access the liver via the portal blood and establish multiple disseminated metastatic hepatic lesions [29]. Preliminary FACS analysis with specific antibodies showed that these cells express αvβ6, but not αvβ8 (Supplemental Fig. S2) and that they can bind **5a**-IRDye680 (Fig. 5A). Immunohistochemical analysis of K8484/GFP/CEA liver metastases with anti-αvβ6 antibodies confirmed the presence of this integrin in tumor tissues (Fig. 5B), whereas imaging studies with **5a**-IRDye800, administered intravenously to tumor-bearing mice, showed efficient accumulation of this conjugate in metastatic colonies in vivo (Fig. 5C). These findings indicate that K8484/GFP/CEA metastatic colonies express αvβ6 in a functionally active form (in terms of peptide **5a** binding). Thus, this model is suitable for in vivo studies on the effect of **5a**-HSA on TGFβ activation.

Tumor-bearing mice were treated with or without **5a**-HSA (50 µg/dose, four treatments for four consecutive days, i. p., *n* = 5 mice/group) (*see* Fig. 5D). The day after the last treatment, the mice were sacrificed, and their livers were explanted for the analysis of TGFβ signaling (pSMAD2/3) in metastases. Microscopic analysis of liver tissue sections showed that all the mice in the control group had metastases (5/5), whereas only 2/5 of the mice in the **5a**-HSA-treated group had liver metastases (Fig. 5E and F). Immunohistochemical analysis of metastatic lesions with anti-pSMAD2/3 antibodies showed a significant reduction in p-SMAD2/3 nuclear localization in the metastases of mice treated with **5a**-HSA, compared with control livers. These findings suggest that **5a**-HSA reduced local TGFβ activation in this model (Fig. 5G and H). Notably, **5a**-HSA did not reduce nuclear p-SMAD2/3 expression in the adjacent non-neoplastic “healthy” liver, indicating that **5a**-HSA could reduce TGFβ activation only in the tumor microenvironment (Fig. 5H).

**Fig. 6:**
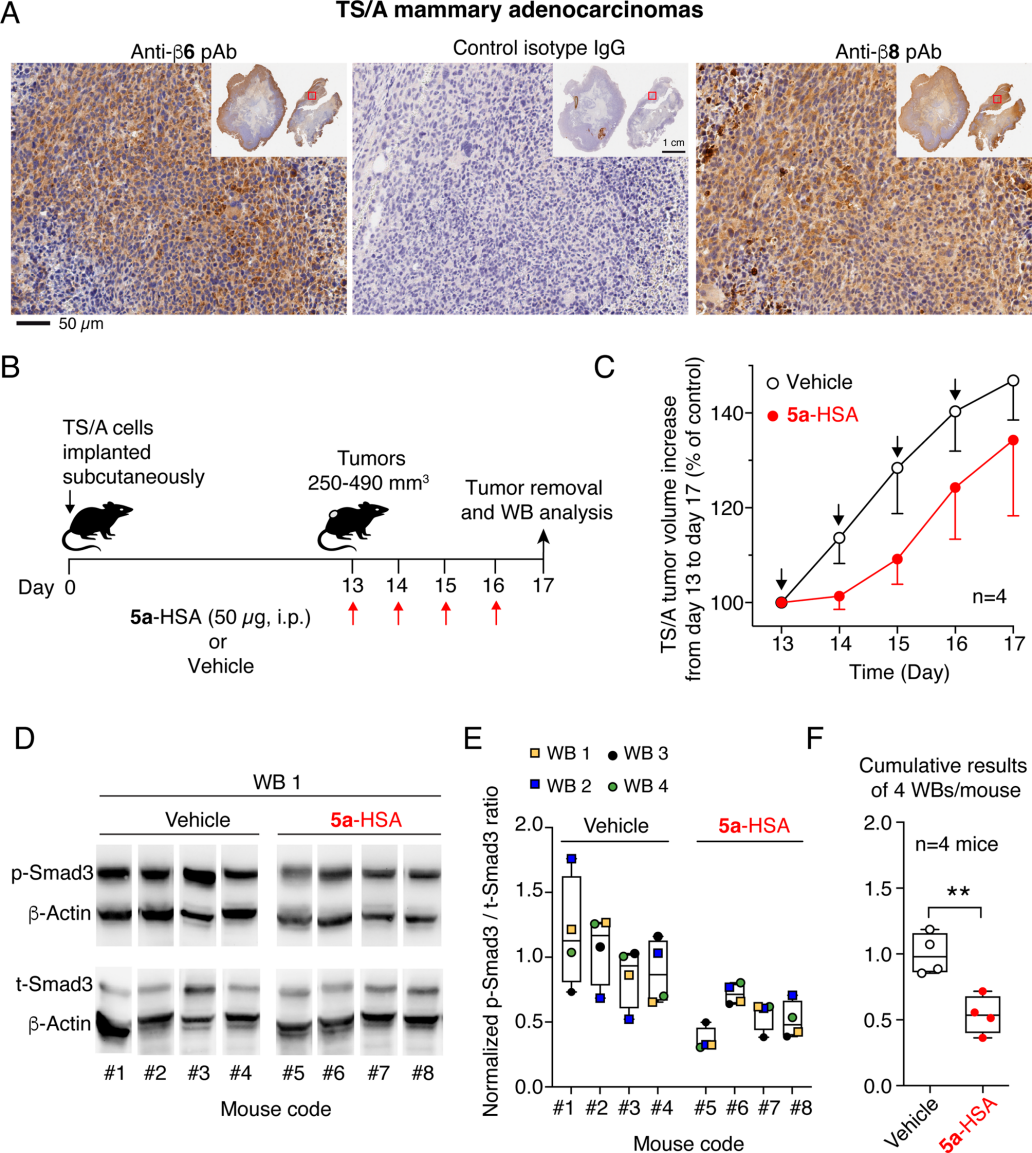
**5a**-HSA inhibits TGFβ signaling in subcutaneous TS/A tumors. **A**) Immunohistochemical analysis of αvβ6 and αvβ8 integrin expression in subcutaneous TS/A tumors as detected by immunohistochemistry using a rabbit anti-β6 or anti-β8 polyclonal antibody followed by an HRP-labelled goat anti-rabbit antibody and DAB substrate (*brown color*). Tumor sections were counterstained with hematoxylin. *Magnification*: 200X. *Inset*: whole tumor slices of two different tumors derived from separate tumor-bearing mice. The red square in the inset outlines the magnified area. **B**-**F**) Effect of **5a**-HSA on tumor growth and TGFβ signaling in mice bearing subcutaneous TS/A tumors, as detected by western blot analysis and quantification of p-SMAD3 and t-SMAD3. (**B**) Experimental scheme. TS/A cells were injected subcutaneously into the left flank. On day 13, mice were randomized into 2 experimental groups. One group (*n* = 4) was treated with **5a**-HSA (50 µg/mouse, i.p., for 4 consecutive days), whereas the other group (*n* = 4) was treated with diluent (*Vehicle*). (**C**) TS/A tumor growth curves. Mean ± SE, *n* = 4 mice. (**D**) One representative western blot analysis, out of four performed, of tumor lysates with antibodies against p-SMAD3, t-SMAD3, and β-actin (as loading control) is shown. **E**-**F**) Quantification of the ratio of normalized p-SMAD3/t-SMAD3 in four independent western blots with results reported separately for each mouse (**D**) or as a cumulative result of the four blots per mouse (**F**). Boxplots with median, interquartile, and 5–95 percentile values. **p < 0.01 by unpaired two-tailed *t-test* analysis

The effect of systemically administered **5a**-HSA on TGFβ activation in the tumor microenvironment was then analyzed in another model based on TS/A mammary adenocarcinoma cells implanted subcutaneously in immunocompetent mice. Although TS/A cells were αvβ6^neg^ and αvβ8^pos(low)^ when cultured in vitro and analyzed by FACS Supplemental Fig. S2A), these cells (expressed both integrins after implantation in mice, as observed by immunohistochemical analysis of tumor tissue sections with specific antibodies (Fig. 6A). **5a**-HSA or its vehicle was administered to tumor-bearing mice (*n* = 4 mice/group, 50 µg/dose, i.p., 4 treatments for 4 days, *see* Fig. 6B and C). One day after the last treatment, the tumors were excised, lysed, and processed for western blot analysis of phospho-SMAD3 using anti-phospho-SMAD3 or anti-total-SMAD3 antibodies (p-SMAD3 and t-SMAD3). Interestingly, a significant reduction in the p-SMAD3/t-SMAD3 ratio was observed in the group of mice treated with **5a**-HSA compared with vehicle-treated mice (Fig. 6D-F), supporting the hypothesis that this conjugate can also reduce TGFβ activation in mammary adenocarcinoma tumors.

### 5a-HSA significantly increases the survival of mice bearing K8484/GFP/CEA pancreatic ductal adenocarcinoma liver metastases

**Fig. 7:**
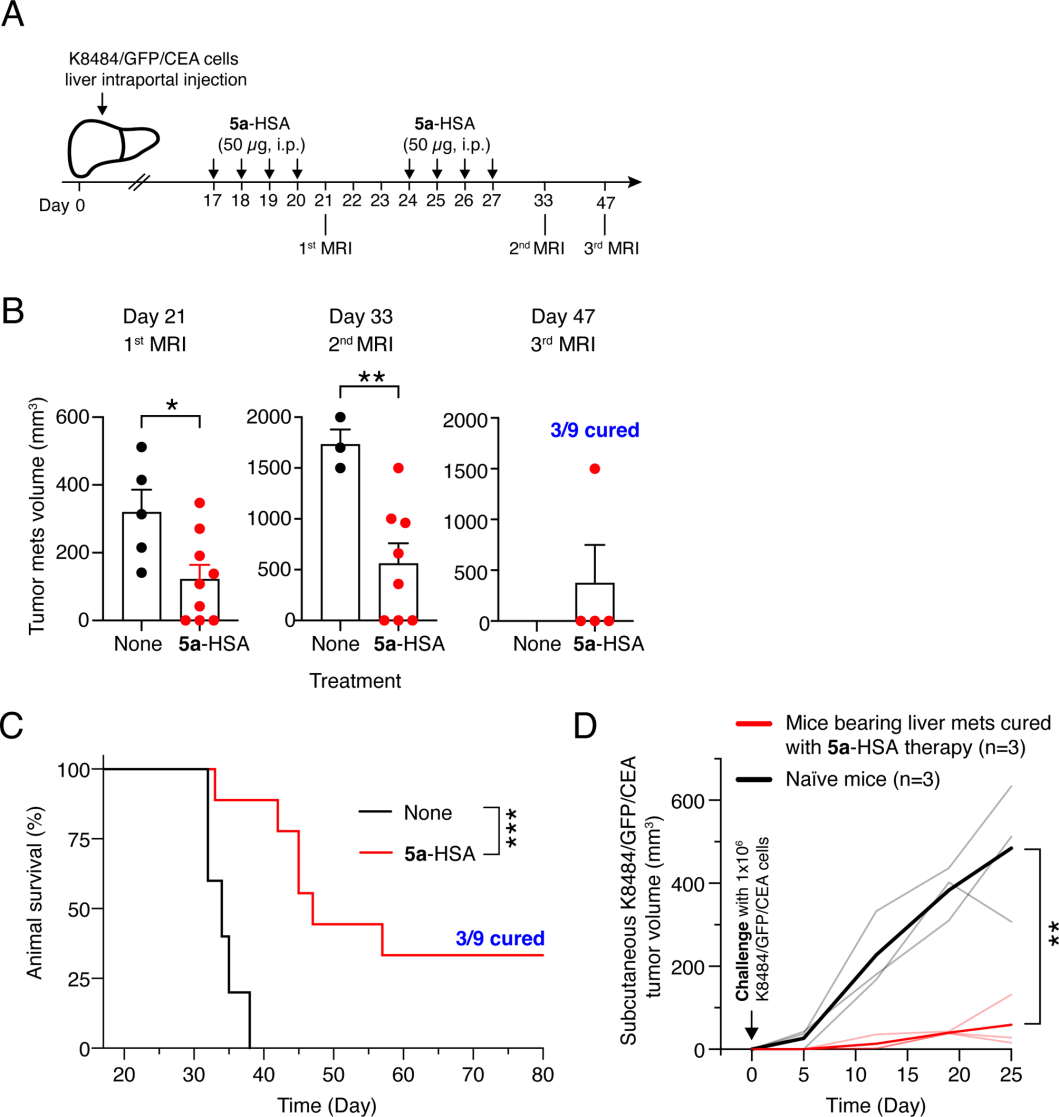
Anti-tumor effect of **5a**-HSA in mice bearing K8484/GFP/CEA liver metastases. **A**) Experimental scheme. K8484/GFP/CEA cells were injected into the portal vein. On day 14, mice were randomized into 2 experimental groups. One group (*n* = 9) was treated with 2 weekly cycles of **5a**-HSA (50 µg/mouse, i.p., for 4 consecutive days per week), whereas the other group (*n* = 5) was left untreated (*None*). **B**) Quantification of the volume of liver mets by MRI at day 21, 33 and 47. *Dots*, total volume of liver mets per mouse. Bar, mean ± SE. *, *P* < 0.05; by unpaired two-tailed *t-test* analysis. Mice were euthanized when the tumor burden reached 2000 mm^3^ or when other signs of distress were observed. **C**) Kaplan-Meier survival curves. ***, *P* < 0.01, by long rank Mantel-Cox test. **D**) Cured mice (*n* = 3) depicted in *panel D* were re-challenged subcutaneously with a tumorigenic dose of K8484/GFP/CEA cells (1 × 10^6^ cells/mouse, at day 120 post-intraportal injection. In parallel, naïve mice (*n* = 3) were challenged subcutaneously with the same cell suspension and served as controls. Tumor growth was monitored by measuring tumor volume using a caliper. Single tumor volume (*thin lines*) and mean tumor volume (*thick lines*) for naïve mice and mice previously cured with **5a**-HSA therapy are shown. **, *P* < 0.05, by unpaired two-tailed *t-test* analysis of the area under the curve for each tumor growth curve

The prolonged half-life of **5a**-HSA and its capability to reduce TGFβ activation in the tumor microenvironment, a potentially important immunosuppressive mechanism, provide a rationale for assessing its therapeutic activity. The anti-tumor efficacy of **5a**-HSA was first investigated in the K8484/GFP/CEA liver metastasis model. Mice with liver metastatic colonies (*n* = 9) were injected with **5a**-HSA (50 µg/mouse, i.p.) for four consecutive days per week, for a total of eight administrations, or were left untreated (*n* = 5) (see Fig. 7A). Tumor growth was then monitored by non-invasive magnetic resonance imaging (MRI) on day 21 (i.e., after the completion of the first cycle of treatment) and again on days 33 and 47, after the second cycle. **5a**-HSA eradicated liver metastases in three out of nine mice (33%) after the first cycle of treatment (Fig. 7B). These mice remained tumor-free on days 33 and 47, whereas the remaining mice showed a significant delay in tumor growth, with an overall median survival time of 47 days (Fig. 7B and C). In contrast, all the control mice (*n* = 5) died between days 32 and 38, with a median survival time of 34 days. Furthermore, a marked delay in tumor growth was observed when the three cured mice were rechallenged (s.c.) with fresh tumor cells compared with the three naïve mice (Fig. 7D).

Notably, the cumulative results of the experiments performed on the K8484/GFP/CEA model, including the experiments reported in the above paragraph (3/5 mice with no metastases in the **5a**-HSA groups) and in this paragraph (3/9 mice with no metastases), show that 6/14 mice could reject tumors (~ 43%) in the **5a**-HSA group, whereas no rejection was observed in the control group.

### 5a-HSA inhibits the growth of subcutaneous lesions of TS/A mammary adenocarcinomas, WEHI-164 fibrosarcomas and TRAMP-C2 prostate adenocarcinomas in mice

**Fig. 8:**
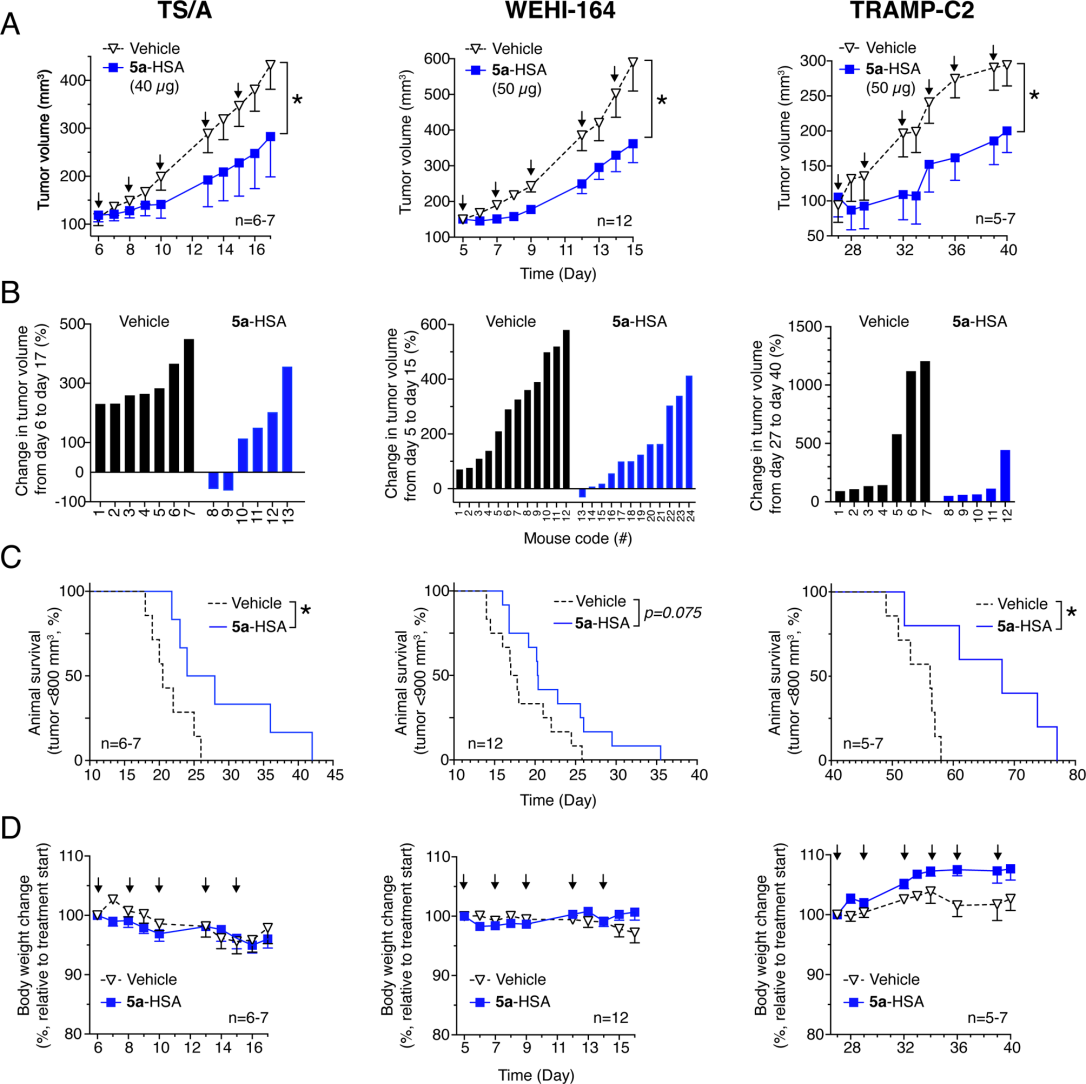
Pharmacological and toxicological effects of **5a**-HSA in mice bearing different tumors implanted subcutaneously. Effect of **5a**-HSA on *(i)* the growth of TS/A mammary adenocarcinomas, WEHI-164 fibrosarcomas, or TRAMP-C2 prostate carcinomas implanted subcutaneously in mice (panels **A** and **B**); *(ii)* animal survival (panels **C**); and *(iii)* body weight (panels **D**). Tumor-bearing mice were treated at the indicated times (*arrows*) with 40–50 µg of **5a**-HSA (i.p.) at the indicated times (arrows). For the WEHI-164 fibrosarcoma model, the cumulative results of two independent experiments (each performed with groups of six mice) are shown. **A**) Graphs of tumor growth (5–12 mice per group, as indicated, mean ± SE). *, *P* < 0.05 by unpaired two-tailed *t-test* analysis of the area under the curve for each tumor growth. **B)** Waterfall plots of percentage change in tumor volume for individual mice over the indicated time. **C**) Kaplan-Meier survival curves (*, *P* < 0.05 by long rank Mantel-Cox test). **D**) Change in body weight (mean ± SE, 5–12 mice)

The anti-tumor activity of **5a**-HSA was then evaluated in other syngeneic subcutaneous tumor models based on TS/A mammary adenocarcinoma, WEHI-164 fibrosarcoma, and TRAMP-C2 prostate carcinoma. Tumors were allowed to grow to 95–150 mm^3^ and then treated with **5a**-HSA (40–50 µg/mouse, i.p.) three times per week for a total of to 5–6 administrations. This conjugate significantly delayed tumor growth in all tested models (Fig. 8 and Supplemental Fig. S5). Notably, in the case of TS/A tumors, **5a**-HSA induced almost complete cancer regression in 2 out of 6 mice, although the tumor recurred after treatment discontinuation (Supplemental Fig. S5). The anti-tumor activity of **5a**-HSA was not associated with signs of toxicity, such as changes in animal body weight, behavior, and aspect of fur.

### The anti-tumor activity of 5a-HSA is inhibited by an anti-CD8 depleting antibody

To assess whether the anti-tumor activity of **5a**-HSA was related to the activation of an immune response, we investigated the effect of **5a**-HSA alone and in combination with an anti-CD8 depleting antibody in the WEHI-164 fibrosarcoma model. The results (Supplemental Fig. S6) showed that the anti-tumor effects of **5a**-HSA were significantly inhibited by the anti-CD8 antibody, but not by an irrelevant antibody used as a negative control, lending support to the hypothesis that inhibition of TGFβ signaling in tumors can unleash an anti-tumor immune response, possibly by reprogramming the tumor immune microenvironment.

### 5a-HSA exerts synergistic anti-tumor effects with S-NGR-TNF

**Fig. 9:**
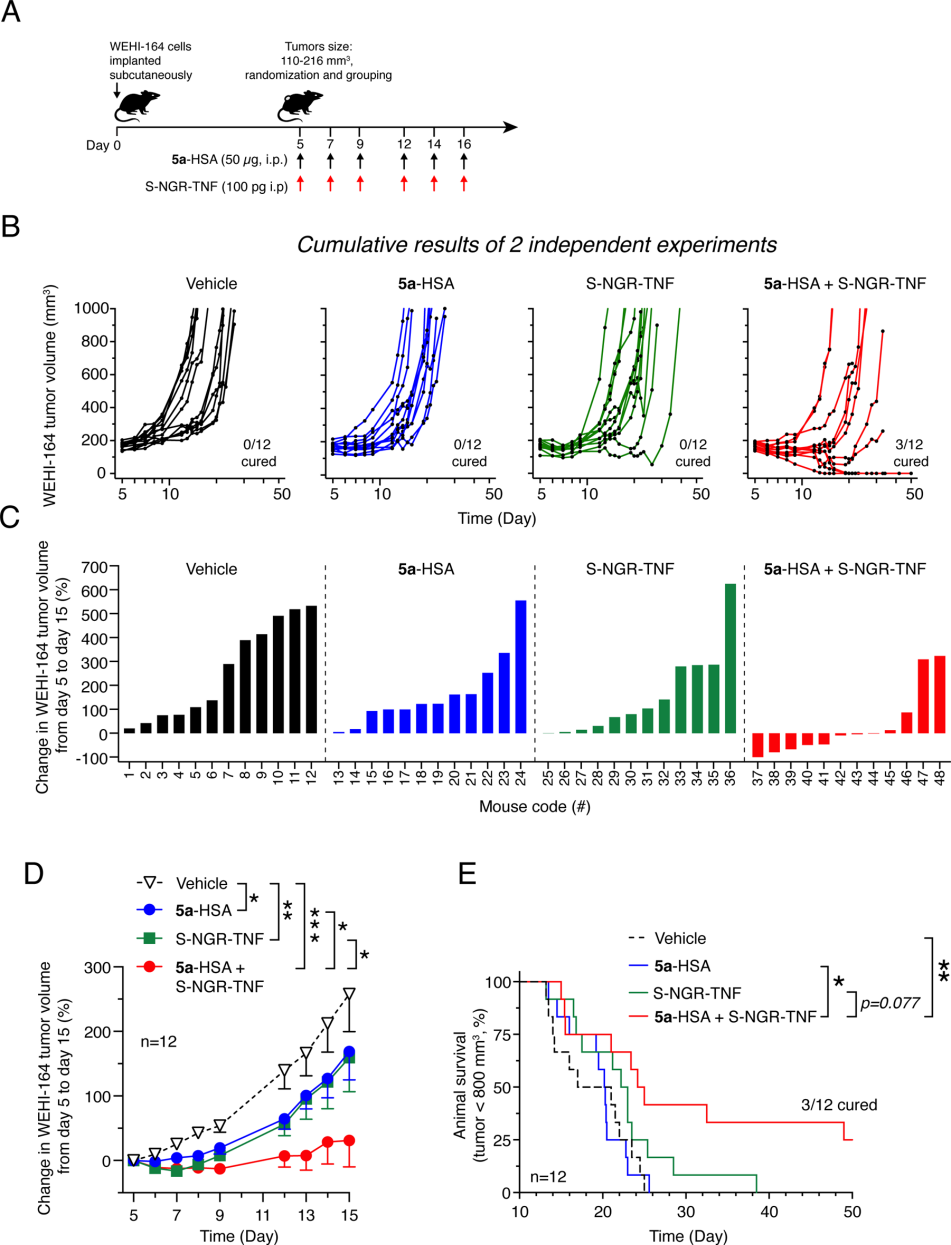
Effect of **5a**-HSA and murine S-NGR-TNF, alone and in combination, on the growth of subcutaneous WEHI-164 tumors. **A**) Experimental scheme. WEHI-164 cells were subcutaneously injected into 12 mice. On day 5 post-implantation, the mice were randomized into 4 experimental groups. Mice were injected intraperitoneally with diluent (*Vehicle*) or with the indicated doses of **5a**-HSA and murine S-NGR-TNF, alone or in combination (at days 5, 7, 9, 12, 14, and 16). Cumulative results of 2 independent experiments are shown (each conducted with 6 mice/group). **B**) Individual tumor growth curves. **C**) Waterfall plots showing the percentage change in tumor volume for individual mice over the indicated time. **D**) Tumor volume (*n* = 12, mean ± SE; *, *P* < 0.05; **, *P* < 0.01, by unpaired two-tailed t-test analysis of the area under the curve for each tumor volume calculated with GraphPad Prism software). **E**) Kaplan-Meier survival curves (*, *P* < 0.05; **, *P* < 0.01, by long rank Mantel-Cox test). Mice were euthanized when the tumors reached 800 mm^3^ or when extensive tumor ulceration or other signs of distress were observed

We then investigated the effect of **5a**-HSA on the anti-tumor activity of S-NGR-TNF, a derivative capable of homing to the tumor vasculature and promoting CD8^+^ T cell infiltration in tumors [34, 35]. The combined treatment of mice bearing subcutaneous WEHI-164 fibrosarcomas (*n* = 12 mice/group) induced stronger anti-tumor effects than those obtained with single compounds (Fig. 9). Notably, while **5a**-HSA and S-NGR-TNF alone were unable to cure mice in this model, the combined treatment cured 3/12 mice with no evidence of toxicity (Fig. 9). These results suggest that **5a**-HSA and S-NGR-TNF exert synergistic effects.

## Discussion

The present study shows that systemic administration of **5a**-HSA can reduce tumor growth in various tumor animal models, including pancreatic ductal adenocarcinoma (PDAC), prostate cancer, mammary adenocarcinoma, and fibrosarcoma. Studies in the fibrosarcoma model have shown that these effects can be inhibited by an anti-CD8 depleting antibody, suggesting that **5a**-HSA promotes an anti-cancer immune response mediated by CD8 T cells. Considering the known capability of peptide **5a** to block αvβ6 and/or αvβ8, i.e. two integrins capable of activating TGFβ-dependent immunosuppressive mechanisms, one possibility is that **5a**-HSA has unleashed an anti-tumor immune response by interfering with this immunosuppressive mechanism and by reprogramming the tumor microenvironment. This view is supported by the observation that TGFβ signaling was reduced in subcutaneous mammary adenocarcinoma and in PDAC metastatic liver lesions after repeated administration of **5a**-HSA to tumor-bearing mice. Interestingly, in vitro studies have shown that peptide **5a** and **5a**-HSA can inhibit the production of TGFβ by various by αvβ6- and/or αvβ8-positive cancer cells (i.e. single or double positive) such as 5M7101/GFP/CEA cells (αvβ6^pos^, αvβ8^neg)^, TS/A mammary adenocarcinoma (αvβ6^neg^, αvβ8^pos(low)^), WEHI-164 fibrosarcoma (αvβ6^neg^, αvβ8^pos(low)^), and TRAMP-C2 prostate carcinoma (αvβ6^neg^, αvβ8^pos^). Furthermore, in vitro studies have shown that peptide **5a** can bind αvβ8^pos^ iTreg cells and impair their ability to induce TGFβ maturation in vitro. Thus, the anti-tumor effects of **5a**-HSA observed in the murine models of pancreatic ductal adenocarcinoma, prostate cancer, mammary adenocarcinoma, and fibrosarcoma may reflect the ability of **5a**-HSA to recognize both αvβ6 and αvβ8 on all these cells, including Tregs, and block their function.

Considering that Treg cells present in tumors can express αvβ8 and contribute to the development of an immunosuppressive microenvironment via TGFβ activation through this integrin, the bi-specific properties of **5a**-HSA may represent an important advantage with respect to mono-specific αvβ6 or αvβ8 integrin ligands previously described in the literature [11, 18, 36–46], as **5a**-HSA can target cancer cells expressing one or both integrins as well as Tregs.

The capability of **5a**-HSA to block αvβ6 and αvβ8 and inhibiting TGFβ activation in tumors might be exploited, in principle, to enhance the anti-tumor effects of therapies aimed at stimulating an immune response. This view is supported by the results showing that **5a**-HSA could enhance, in the WEHI-164 fibrosarcoma model, the anti-tumor activity of S-NGR-TNF, a targeted inflammatory cytokine consisting of tumor necrosis factor-alpha (TNF) fused to a tumor vasculature-homing peptide containing the NGR sequence [21]. Considering the known antagonistic cross-talk between tumor necrosis factor-alpha (TNF) and TGFβ in cancer [47], the reduction of TGFβf in the tumor microenvironment caused by **5a**-HSA might have contributed to the synergism observed between **5a**-HSA and S-NGR-TNF in mice.

## Conclusion

**5a**-HSA can be used as a drug to block αvβ6- and αvβ8-dependent TGFβ activation in tumors and to promote immunotherapeutic responses. These findings may prompt further investigations aimed at assessing the therapeutic value of **5a**-HSA combined with S-NGR-TNF in other models, as well as in combination with other immunostimulatory or immunotherapeutic agents, such as cytokines, immune checkpoint inhibitors, CAR-T cells, cytotoxic lymphocytes, or other cells of the innate and adaptive immune system, in models characterized by different expression levels of αvβ6 and αvβ8 integrins.

## Electronic supplementary material

Below is the link to the electronic supplementary material.

Supplementary Material 1

## Data Availability

All the data generated or analyzed during this study are included in this published article and its supplementary information files. Raw images are available in the San Raffaele Open Research Data repository https://ordr.hsr.it/research-data/. Further information and requests for resources should be directed to Flavio Curnis (curnis.flavio@hsr.it).

## Abbreviations

CgA: Chromogranin A
TGFβ: Transforming growth factor-β
LAP: Latency-associated peptide
MS: Mass spectrometry
MRI: Magnetic resonance imaging
