# Supplementary material for: Blockade of αvβ6 and αvβ8 integrins with a chromogranin A-derived peptide inhibits TGFβ activation in tumors and suppresses tumor growth
Source: J Exp Clin Cancer Res. 2025 Mar 8;44:88. doi: 10.1186/s13046-025-03352-4 (PMC11889887; doi:10.1186/s13046-025-03352-4)
Supplement: Supplementary file 1 — Supplementary Material 1 [file 13046_2025_3352_MOESM1_ESM.docx]

**Supporting Information**

**Blockade of αvβ6 and αvβ8 integrins with a chromogranin A-derived peptide inhibits TGFβ activation in tumors and suppresses tumor growth**

**Supplemental Tables**

**Supplemental Table S1. Binding affinity of human αvβ6 and αvβ8 integrins for peptide 5a and 5a-HSA.**

| **Code** | **Compound** ^a^ | ***Ki*** ^b^  (by competitive  integrin binding assay, nM) | | |  | ***Kd* ^c^**  (by direct integrin  binding assay, nM) | | |
| --- | --- | --- | --- | --- | --- | --- | --- | --- |
|  |  | αvβ6 |  | αvβ8 |  | αvβ6 |  | αvβ8 |
| **5a** | CFETLRGD**L**RILSILR**X_1_**QNL**X_2_**KELQD_-CONH2_ | 1.70 ± 0.26 |  | 3.90 ± 1.31 |  | *NA* ^d^ |  | *NA* |
| **5a**-HSA | **5a**-human serum albumin conjugate | 0.102 |  | 1.04 |  | 0.113 |  | 0.62 ± 0.18 |

a) Single letter code peptide sequence; *-CONH2*, C-terminal amidated; triazole bridge between residues ***X_1_*** and ***X_2_*** (propargylglycine and azidolysine, respectively).

b) *Ki*, inhibition constant. Mean ± SE.

c) *Kd,* apparent dissociation constant*.* Mean ± SE.

d) *NA*, not applicable.

**Supplemental Table S2. Plasma half-life of peptide 5a and 5a-HSA.**

| **Code** | **Administration**  **route** | **Mouse**  **strain** | **Injected**  **dose**  **(nmol)** | **Plasma**  **half-life**  **(min, (h))** |
| --- | --- | --- | --- | --- |
| **5a** ^a^ | Intravenous | BALB/c | 1.2 | 8 min (0.133 h) |
|  |  |  |  |  |
| **5a**-HSA | Intravenous | C57BL/6 | 0.715 | 100 min (1.66 h) |
|  | Intraperitoneal | C57BL/6 | 0.715 | 960 min (16 h) |

1. Coupled to IRDye800 for spectrofluorometric detection.

**Supplemental Figures**


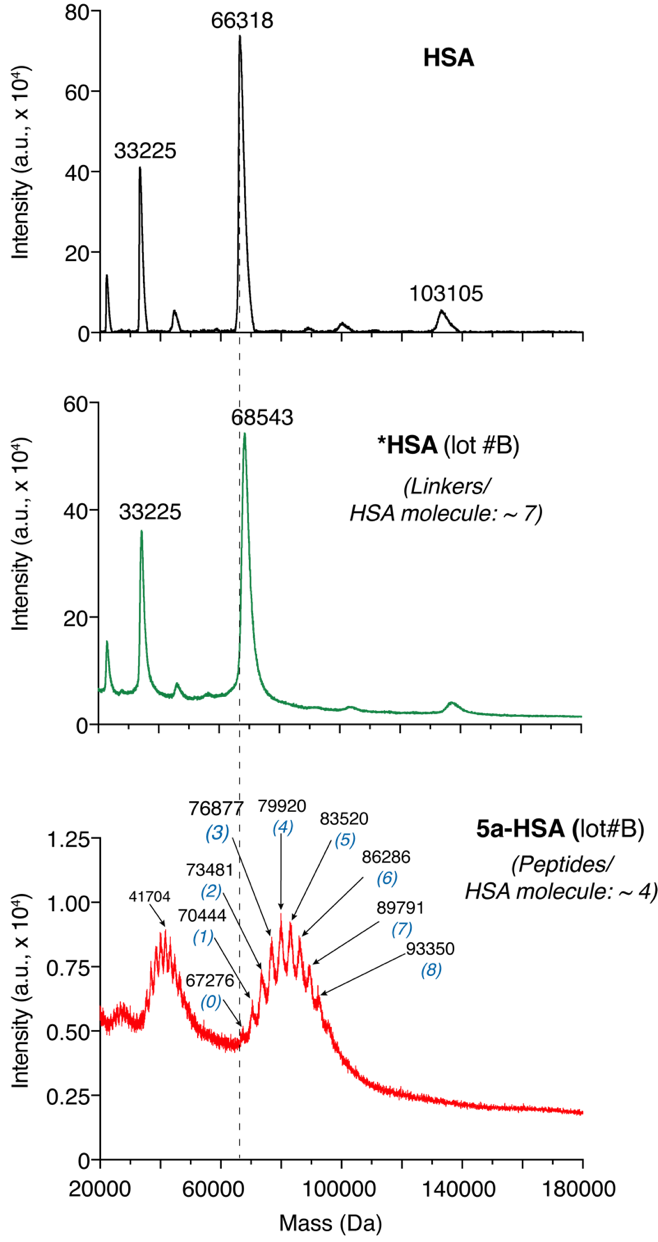


**Supplemental Figure S1. Biochemical characterization of 5a-HSA (Lot #B).**

MALDI-TOF mass spectra of HSA, *HSA, and **5a**-HSA (Lot #B). The average masses of the principal components and the estimated number of peptides coupled to HSA molecules are indicated.


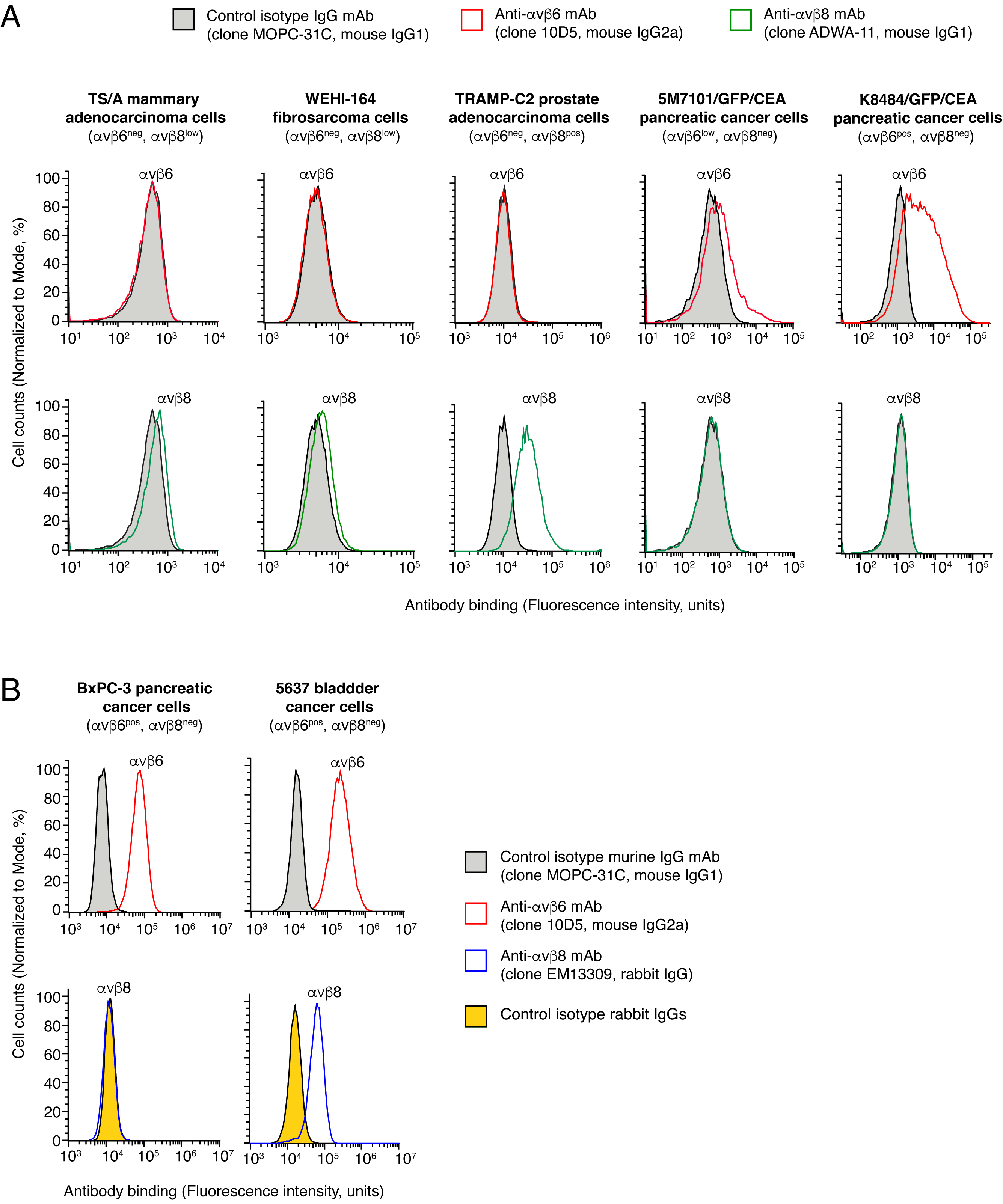


**Supplemental Figure S2. Flow cytometry analysis of αvβ6 and αvβ8 expression in TS/A, WEHI-164, TRAMP-C2, 5M7101/GFP/CEA, K8484/GFP/CEA, BxPC-3 and 5637 cells.**

Expression of αvβ6 and αvβ8 in murine (**A**) and human (**B**) cell lines. Flow cytometry was performed as described previously ^20^ using the indicated monoclonal antibodies, followed by Alexa Fluor 488-conjugated goat anti-mouse, Alexa Fluor 488-conjugated goat anti-rabbit IgG polyclonal antibody, or an Alexa Fluor 647-conjugated goat anti-mouse polyclonal antibodies (only for 5M7101/GFP/CEA and K8484/GFP/CEA).

**
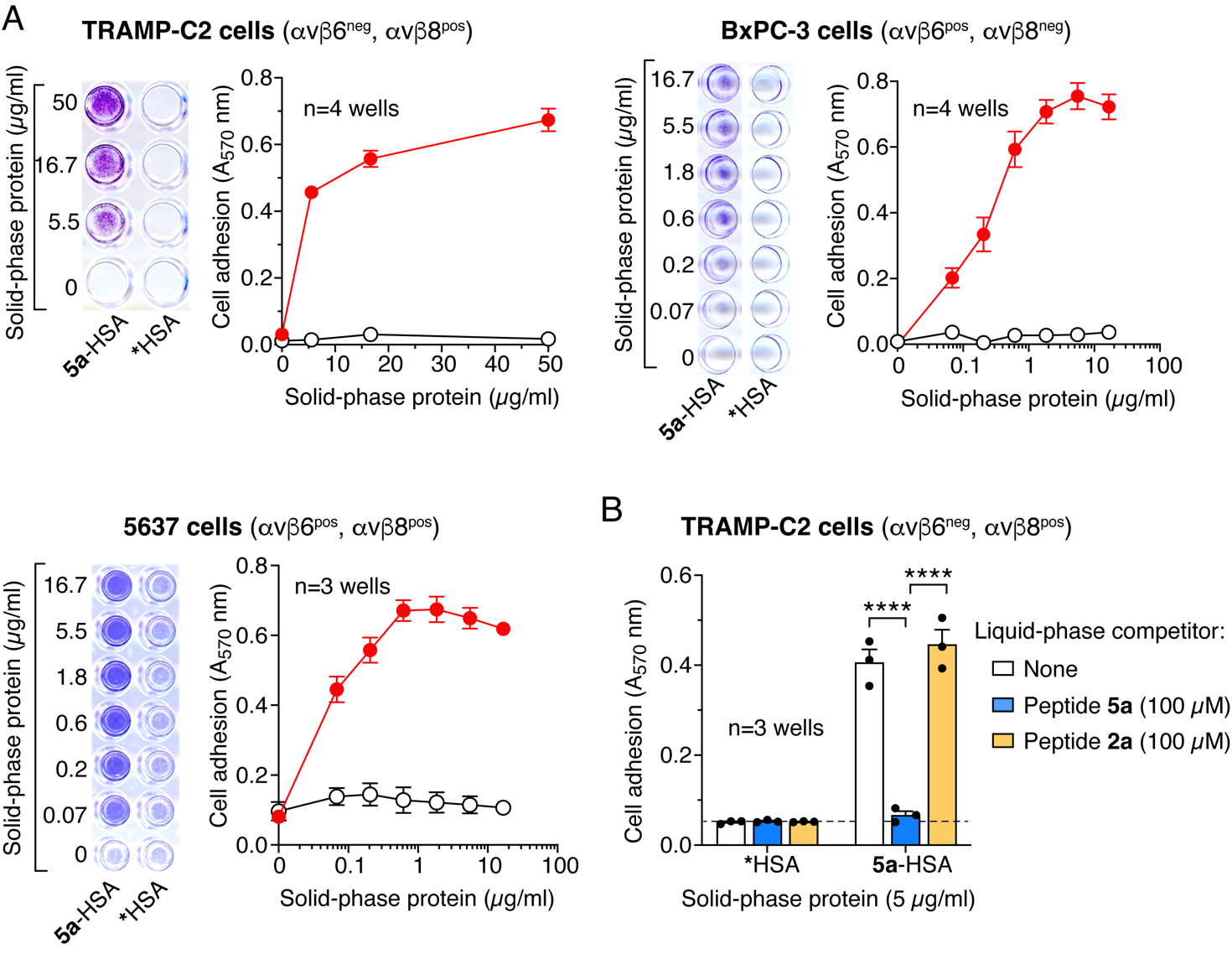
**

**Supplemental Figure S3. 5a-HSA promotes the adhesion of αvβ6/αvβ8 single- or double-positive TRAMP-C2, BxPC-3, and 5637 cell lines*.***

Ninety-six-well PVC microplates were coated with **5a**-HSA or with *HSA, a control conjugate without peptide **5a,** and seeded with the indicated cell lines (0.5-1x10^5^ cells/well). After 2-3 h of incubation, non-adherent cells were removed by washing, and adherent cells were stained with crystal violet and quantified by spectrophotometric analysis using an A_570 nm_ filter (iMark Microplate Absorbance Reader, Bio-Rad). In cases of uneven cell adhesion, microtiter plates were treated with 10% acetic acid (150 µL/well) to dissolve crystal violet, and the absorbance of each well was quantified as described above.

**A**) Representative photomicrographs of wells coated with **5a**-HSA or *HSA; quantification of cell adhesion is shown (mean±SE, n=3-4 wells).

**B**) Effect of peptide **5a** and **2a** (a control peptide with the RGE sequence instead of RGD) on the adhesion of TRAMP-C2 cells to microtiter plates coated with **5a**-HSA or *HSA.

**
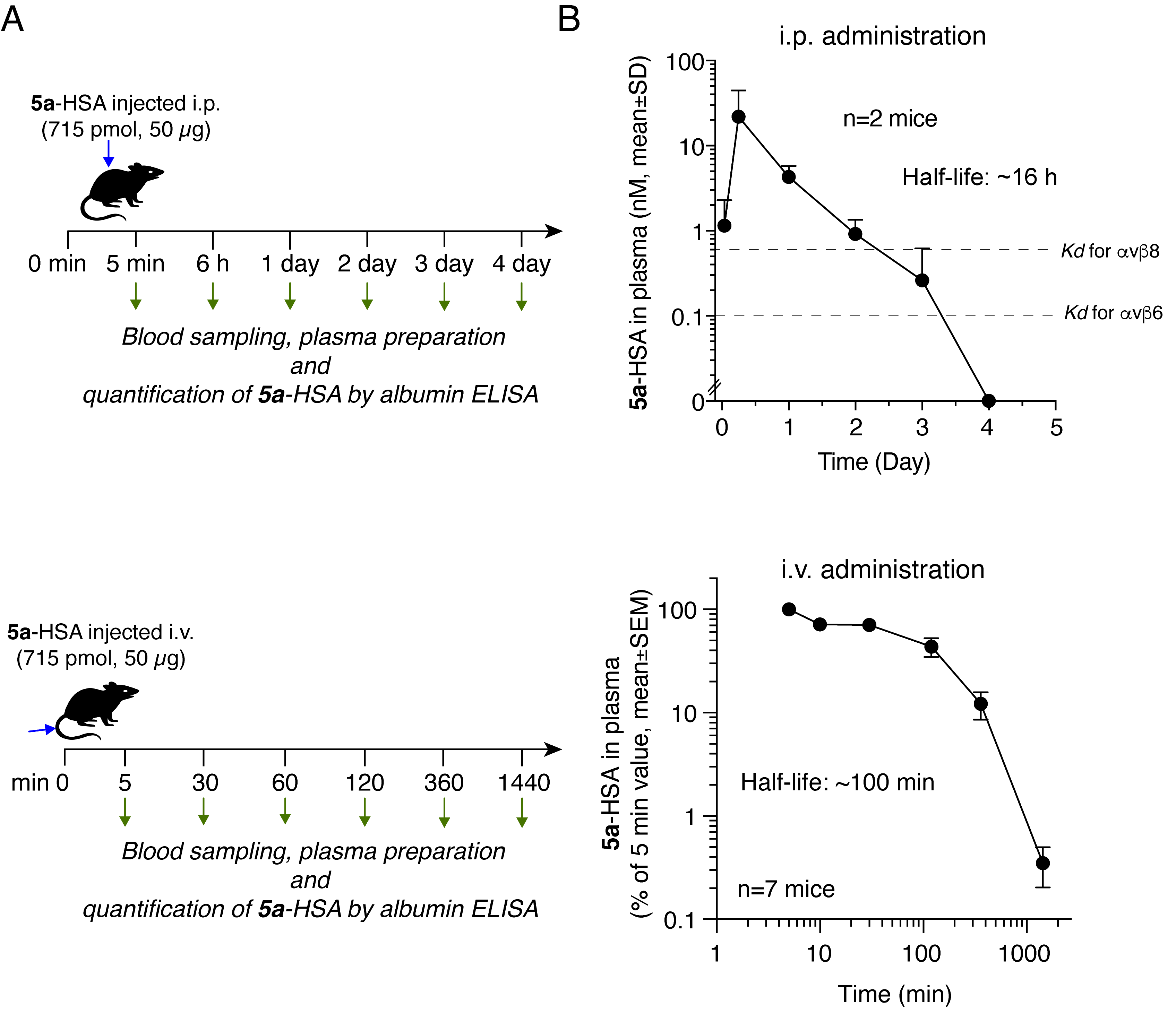
**

**Supplemental Figure S4. Pharmacokinetics of 5a-HSA in mice.**

**A**) Scheme of **5a**-HSA administration and blood sampling in healthy mice. BALB/c and C57BL/6 mice were used for intraperitoneal (i.p.) and intravenous (i.v) pharmacokinetic studies, respectively.

**B)** Plasma levels of **5a**-HSA. Plasma levels of **5a**-HSA were measured after i.p. or i.v. administration using a homemade sandwich-ELISA for human serum albumin and **5a**-HSA as the reference standard.

Dashed lines indicate *Kd* of **5a**-HSA for αvβ6 and αvβ8 integrins.

The plasma half-life of **5a**-HSA was calculated using a one-phase exponential decay equation (GraphPad Prism software, San Diego, California, USA).

**
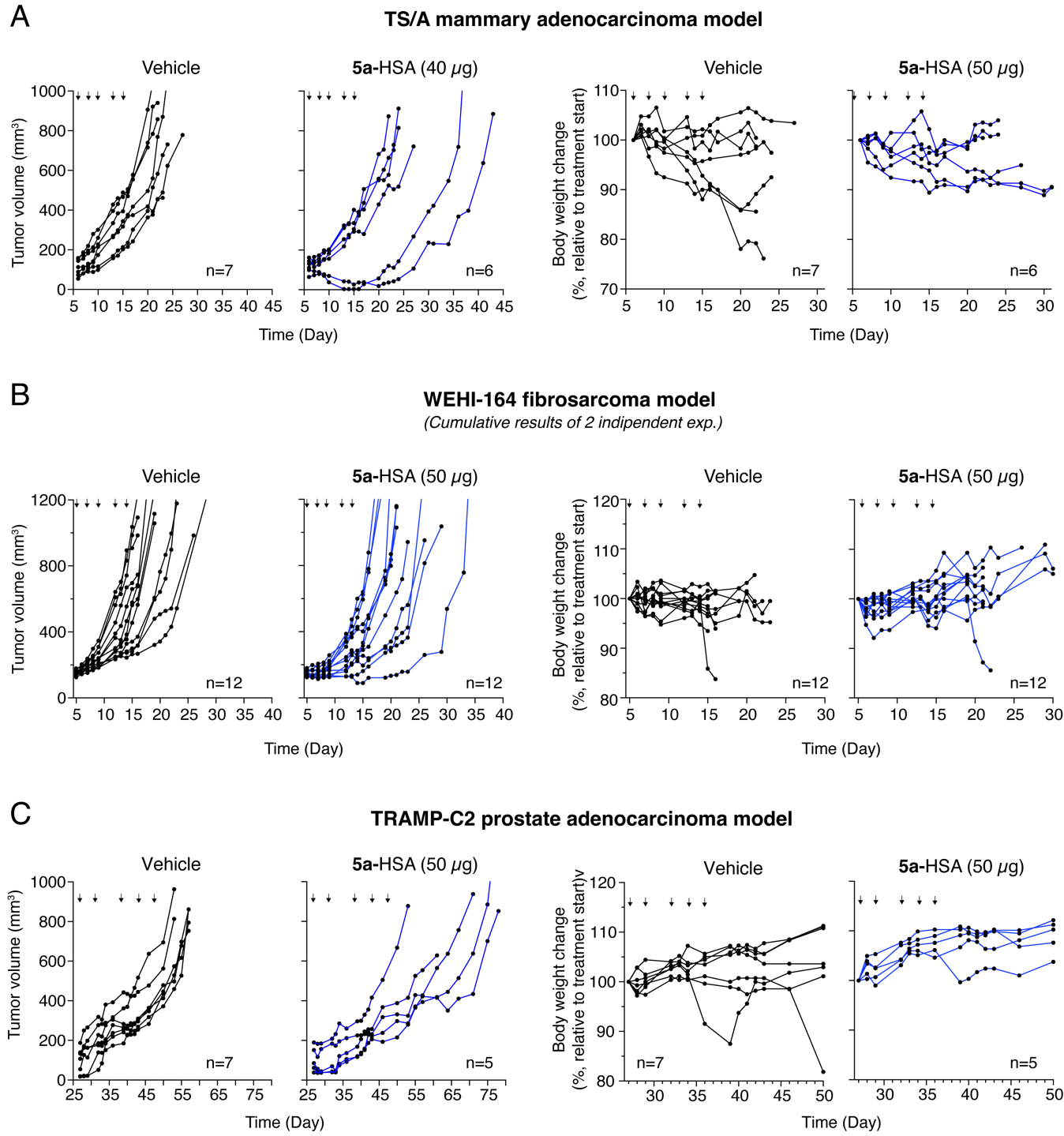
**

**Supplemental Figure S5. Pharmacological and toxicological effects of 5a-HSA in tumor-bearing mice.**

Individual tumor growth curves and changes in the body weights of the mice are shown in **Figure 8**. Mice were euthanized when tumors reached 1000-1200 mm³ or when extensive tumor ulceration or other symptoms were observed


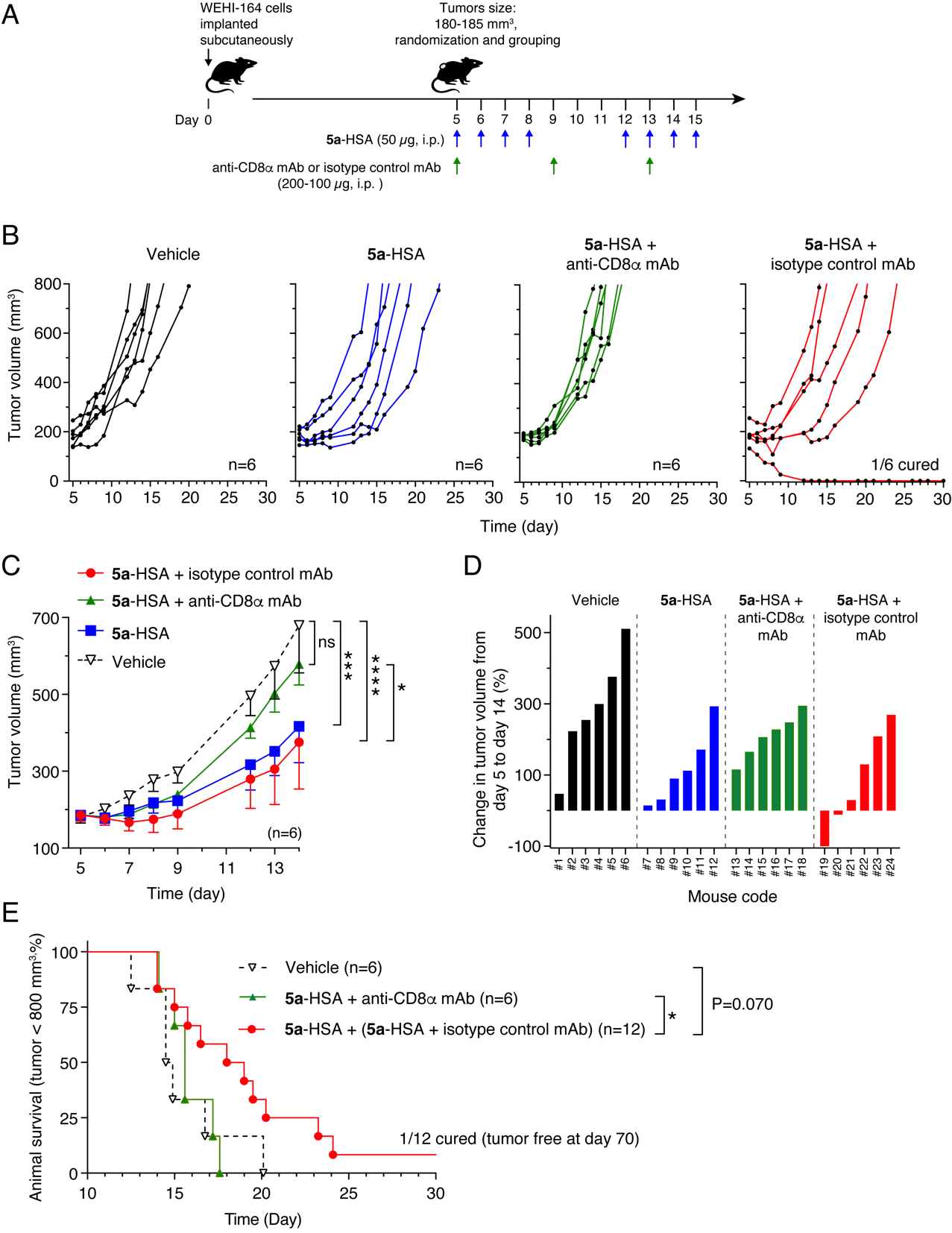


**Figure S6. The administration of anti-CD8α antibodies neutralizes the anti-tumor activity of 5a-HSA against subcutaneous WEHI-164 fibrosarcomas.**

**A**) Scheme of the experiment. WEHI-164 tumor-bearing mice (6 mice/group) were injected (i.p.) with 200 µg (day 5 and 9) or with 100 µg (day 13) of an anti-CD8α monoclonal antibody. Ten minutes later, the mice were injected i.p. with 50 µg of **5a-**HSA. On the following days (days 6, 7, 8, 12, 14, and 15), the mice were treated with **5a**-HSA. **B**) Individual tumor growth curves. **C**) Tumor volume (mean±SE). *, P<0.05; ***, P<0.001, ****, P<0.0001 by two-way ANOVA with post-hoc Tukey's multiple comparisons test. **D**) Waterfall plots of the percentage change in tumor volume for individual mice over the indicated time. **E**) Kaplan-Meier survival curves. Mice were euthanized when the tumors reached >800 mm^3^ or when extensive tumor ulceration or other signs of distress were observed. The cumulative data for **5a**-HSA with **5a**-HSA + isotype control mAb are reported. *P < 0.05; by log-rank (Mantel-Cox) test.
